# An in vitro model for studying CNS white matter: functional properties and experimental approaches

**DOI:** 10.12688/f1000research.16802.1

**Published:** 2019-01-29

**Authors:** Silvia Bijland, Gemma Thomson, Matthew Euston, Kyriakos Michail, Katja Thümmler, Steve Mücklisch, Colin L. Crawford, Susan C. Barnett, Mark McLaughlin, T. James Anderson, Christopher Linington, Euan R. Brown, Eric R. Kalkman, Julia M. Edgar

**Affiliations:** 1 Institute of Infection, Immunity and Inflammation, College of Medical Veterinary and Life Sciences, University of Glasgow, Glasgow, G12 8TA, UK; 2 Institute of Biological Chemistry, Biophysics and Bioengineering, Heriot Watt University, Edinburgh, EH14 4AS, UK; 3 School of Veterinary Medicine, College of Medical Veterinary and Life Sciences, University of Glasgow, Glasgow, G61 1QH, UK; 4 Institute of Cancer Sciences, College of Medical Veterinary and Life Sciences, University of Glasgow, Glasgow, G61 1QH, UK

**Keywords:** (re)myelination, microglia, microelectrode array, semi-high throughput, spinal cord, siRNA

## Abstract

The normal development and maintenance of CNS white matter, and its responses to disease and injury, are defined by synergies between axons, oligodendrocytes, astrocytes and microglia, and further influenced by peripheral components such as the gut microbiome and the endocrine and immune systems. Consequently, mechanistic insights, therapeutic approaches and safety tests rely ultimately on *in vivo* models and clinical trials. However, *in vitro* models that replicate the cellular complexity of the CNS can inform these approaches, reducing costs and minimising the use of human material or experimental animals; in line with the principles of the 3Rs. Using electrophysiology, pharmacology, time-lapse imaging, and immunological assays, we demonstrate that murine spinal cord-derived myelinating cell cultures recapitulate spinal-like electrical activity and innate CNS immune functions, including responses to disease-relevant myelin debris and pathogen associated molecular patterns (PAMPs).  Further, we show they are (i) amenable to siRNA making them suitable for testing gene-silencing strategies; (ii) can be established on microelectrode arrays (MEAs) for electrophysiological studies; and (iii) are compatible with multi-well microplate formats for semi-high throughput screens, maximising information output whilst further reducing animal use. We provide protocols for each of these. Together, these advances increase the utility of this *in vitro* tool for studying normal and pathological development and function of white matter, and for screening therapeutic molecules or gene targets for diseases such as multiple sclerosis, motor neuron disease or spinal cord injury, whilst avoiding *in vivo* approaches on experimental animals.

Research highlights **Scientific benefits:**

- Recapitulates in vivo-like myelination, innate immune responses and neuronal electrical activity and is amenable to live cell imaging.
- Compared to single cell type or mixed PNS-CNS cell cultures, this pure CNS multi-cell type system more closely resembles the *in vivo* situation.
- Uncouples CNS-intrinsic responses from those mediated by peripheral organs and systems.
- Ease of manipulation, i.e. genetic using siRNA; neuronal electrical activity using pharmacological modulators of neural activity; small molecules using small molecule libraries; relevant PAMPs or DAMPS.
- Can be generated from transgenic reporter mice for live imaging and functional readouts or from mutant or transgenic models of disease.

**3Rs benefits:**

- Inform studies and/or acts as an adjunct to studies using *in vivo* models of multiple sclerosis, motor neurone disease, the leukodystrophies and other neurodegenerative diseases involving white matter, minimising the use of experimental animals and maximising information obtained.

**Practical benefits:**

- Straightforward to establish in any lab with cell culture facilities.
- Compared to CNS slice cultures, which also contain all major neural cell types, this cell culture system is easier to maintain (oxygen and nutrients readily reach all cells) and quicker to set up.
- Cells can be grown on microelectrode arrays that do not require the specialist expertise needed for single cell electrophysiology.
- Cells can be grown on multi-well plates for semi-high throughput assays.
- Multi-well microplate formats facilitate the testing of multiple factors on parallel cultures.

**Current applications:**

- Semi-high throughput screens for pro-myelinating or inhibitory factors; testing and verifying siRNA constructs and/or testing the consequences of gene knockdown; modulating neural activity to assess secondary consequences for other cells types; live imaging of cellular interactions.

**Potential applications:**

- Semi-high throughput screens for factors that affect axonal survival or regeneration.
- Assessing effects of microglial ablation on other cell types, by manipulating CSF1R signalling pathway.
- Live imaging of organelle distribution and/or transport.
- Initial screen of anti-sense oligonucleotides for gene silencing in the context of *in vivo*-like cellular phenotypes and morphologies.

## Introduction

Diseases that affect white matter are many and varied, and include spinal cord injury, motor neuron disease, Alzheimer’s and multiple sclerosis. Typically, these diseases are modelled and studied in experimental rats and mice, and occasionally in primates, undergoing procedures such as spinal cord contusion, genetic modification or induction of experimental allergic encephalopathy (EAE); procedures that range in severity from moderate to severe under Animals (Scientific Procedures) Act 1986 licensing. Whilst such studies can and do provide important information relevant to human disease, they require careful design and large numbers of animals to provide sufficient power ( Baker & Amor, 2014). For example, we estimate that to test the efficacy of a single drug, 3 cohorts each of 12 animals with EAE is required, depending on effect size and consistency in response (C. Linington, personal communication). Alternative approaches include *in vitro* techniques, such as human-derived induced pluripotent stem cell models or murine cell cultures.

Indeed, cell and tissue culture is used widely in neuroscience to study the development and function of the major cell types of the CNS; neurons, oligodendroglia, astrocytes and microglia. The main reasons being, *in vitro* models are a) relatively inexpensive; b) amenable to manipulation, including pharmacological and genetic; c) accessible to live imaging and optogenetic approaches; d) reduce the reliance on experimental animal models for early-stage screening/proof-of-concept studies; and e) inform subsequent animal studies, if they are required. In particular, cell culture assays can reduce the numbers of animal used in *in vivo* studies by, for example (i) guiding selection of candidate therapeutic molecules, (ii) informing drug dosage concentrations, (iii) measuring toxicity (iv) defining gene targeting efficiency (v) testing gene constructs prior to the generation of transgenic animals.

Single cell type-enriched cultures have been invaluable in addressing questions relating to cell autonomous characteristics ( Bechler *et al*., 2015; Lee *et al*., 2012a; Mei *et al*., 2014; Sanchez-Gomez *et al*., 2018) and simple co-cultures have shed light on bi-cellular interactions ( Froger *et al*., 2010; Lundgaard *et al*., 2013). However, multi-cell type ( Madhavan *et al*., 2018; Thomson *et al*., 1993) or explant cultures ( Thomson *et al*., 2006; Zhang *et al*., 2011) that maintain the complex cellularity and functional properties of the CNS, more closely represent the *in vivo* situation. As such, they are more relevant for addressing questions where the answer relies on physical interactions and/or paracrine signaling between CNS cell types; notwithstanding they remain uncoupled from the influence of peripheral organs or systems including the gut microbiome, the adaptive immune system and the endrocrine system.

Myelinated nerve fibers are the culmination of complex bidirectional molecular, structural and functional interactions between axons and oligodendrocytes. For example, neuronal electrical activity modulates myelination during development ( Demerens *et al*., 1996; Wake *et al*., 2011 and reviewed in Almeida, 2018; Almeida & Lyons, 2017; Zalc & Fields, 2000) and the myelinating cell supports the myelinated axon throughout life ( Edgar *et al*., 2009; Griffiths *et al*., 1998; Lappe-Siefke *et al*., 2003) by modulating axonal transport and the axonal cytoskeleton ( Edgar *et al*., 2004; Kirkpatrick *et al*., 2001; Pan *et al*., 2005), maintaining white matter homeostasis ( Kassmann *et al*., 2007; Schirmer *et al*., 2018) and providing energy substrates ( Fünfschilling *et al*., 2012; Lee *et al*., 2012b; Meyer *et al*., 2018). Adjacent microglia and astrocytes shape and support the myelinated fiber during development and adulthood (reviewed in Allen & Lyons, 2018; Thion *et al*., 2018). For example, microglia clear apoptotic oligodendrocytes generated in excess during development ( Barres & Raff, 1994; Barres *et al*., 1992), boost developmental myelination by expressing IGF-1 ( Wlodarczyk *et al*., 2017) and remove debris following demyelination or Wallerian degeneration, albeit slowly ( George & Griffin, 1994). Astrocytes secrete factors that enhance developmental myelination ( Ishibashi *et al*., 2006 and reviewed in Barnett & Linington, 2013), form gap junctions with each other and with oligodendrocytes for exchange of ions and small metabolites (reviewed in Cotrina & Nedergaard, 2012) and regulate the structure of the mature myelin sheath through secretion of inhibitors of thrombin proteases ( Dutta *et al*., 2018). Conversely, ‘activated’ microglia and astrocytes can contribute to disease pathogenesis and injury to myelinated fibers, as in mouse models of motor neuron disease ( Beers *et al*., 2006; Boillee *et al*., 2006; Hall *et al*., 1998; Nagai *et al*., 2007; Yamanaka *et al*., 2008), the leukodystrophies ( Ip *et al*., 2007), and cerebral vascular disease ( Fowler *et al*., 2018). Thus, *in vitro* models that replicate the interdependence of cells of the intact CNS provide an informative prelude or adjunct to *in vivo* studies.

We previously described a murine, spinal cord-derived myelinating cell culture system ( Thomson *et al*., 2006; Thomson *et al*., 2008) to explore axonal organelle distribution ( Edgar *et al*., 2008), cell dynamics ( Ioannidou *et al*., 2012), pro-myelination factors ( Berghoff *et al*., 2017; Goebbels *et al*., 2017) and neurotropism and pathogenesis of Zika virus infection ( Cumberworth *et al*., 2017). However, little is known about the culture’s functional properties with respect to neuronal electrical activity or innate immune responses; factors that influence normal development as well as pathology. Further, the possibility of modifying it for use as an electrophysiological or gene silencing assay has not been explored. Here we validate this system as a functional model of CNS white matter, and describe adaptations to increase its utility in the study of development and disease. This model is straightforward to establish in any laboratory with basic cell culture facilities, and its use can be extended as described here, if equipment is available for live imaging, microelectrode array and/or multi-well plate microscopy.

## Methods

### Mice

All animals were bred and maintained in conventional caging, with up to 4 cage companions, in the Biological Services Facilities (BSF) at the University of Glasgow; 12 h light-dark cycle and food and water *ad libitum*. Bedding was non-sterile wood-chip; food was normal maintenance diet; water was normal tap water; temperature was 19–21 °C; and humidity was 55% ± 10%. Environmental enrichment took the form of mini-tubes, sizzle nest and burrowing treats (sunflower and pumpkin seeds). The mice themselves were not health-screened but the BSF was free of the major rodent pathogens but positive for some adventitious agents, namely – for the period in question – pinworms, *Helicobacter spp*., *Pasteurella pneumotropica* and Mouse Norovirus. Embryonic day 13 (E13; day of plug being E0) mice (of both sexes; sex undetermined) were obtained by time mating wild type mice; *Cnp* ^+/+^ with *Cnp* ^-/-^ mice ( Lappe-Siefke *et al*., 2003), both on a C57BL/6J (Charles River) background; or wild type females with hemizygous *Thy1*-CFP males ( Feng *et al*., 2000), both on a C57BL/6N (Charles River) background. Pregnant dams were killed on the morning of E13 by cervical dislocation followed by decapitation and the uterine horns containing the developing embryos were removed after laparotomy, and immediately placed on ice in a sterile 10 cm Petri dish. Adult (postnatal day [P] 60 – P120, male and female homozygous *Plp1* transgenic (line #72; ( Readhead *et al*., 1994) and wild type mice (littermates or closely related mice from the same colony) were killed in gradually increasing levels of CO _2_, followed by decapitation, and the spinal cord was extracted rapidly for preparation of a myelin-enriched tissue fraction. All animal use was approved by the Ethical Committee of the University of Glasgow and licensed under the Animal [Scientific Procedures] Act 1986 project licence PPL60/3656. Experiments on animal-derived cell cultures were conducted according to ARRIVE guidelines, including randomisation of samples in multi-well microplates, blinding of the experimenter and/or automation of quantification, as indicated. Numbers of technical repeats and independent biological repeats are indicated in the Figure Legends. Technical repeat: independent wells/dishes from a single cell culture. Biological repeat (or experimental unit): independent cell culture, generated by pooling all embryos from a single pregnant mouse.

*Plp1* tg mice ≥3 months of age mice are prone to seizures. Mice appearing dull or apathetic (suggesting a post-ictal problem) or which has an observed seizure lasting more than 2 minutes were killed by a humane method. In the vast majority of instances, the mice were used prior to the development of seizures.

### Myelinating cell cultures

A step-by-step protocol is provided in the supplementary protocol document ( Supplementary File 1). The procedure for preparing murine myelinating cultures described ( Thomson *et al*., 2008) was modified slightly. Briefly, E13.5 mouse spinal cords were dissected, then incubated in 1 ml per 6 cords of 0.25% trypsin in HBSS minus calcium and magnesium for 15 minutes at 37°C. The digestion was stopped using 1 ml per 6 cords SD solution ( Thomson *et al*., 2008) or Plating Medium (PM; 50% DMEM, 25% HBSS and 25% horse serum) plus 0.04 mg/ml DNase (or more, if required). The cells were triturated, resuspended in PM and plated on coverslips (3 x 13 mm diameter per 35 mm Petri dish), imaging dishes or microelectrode arrays (MEAs) at ~150,000 cells per 100 μl PM, per 133 mm ^2^; or in 96 or 384-well dishes in 50 μl PM, at various concentrations (see Results). From day *in vitro* (DIV) 0, cells were grown in 50% PM and 50% differentiation medium (DMEM supplemented with 30% D-glucose (4500 mg/l glucose final), 10 ng/ml biotin, 50 n M hydrocortisone, 10 μg/ml insulin, 0.5% hormone mix (stock concentration 1 mg/ml apo-transferrin, 20 m M putrescine, 4 μ M progesterone, and 6 μ M selenium; based on ( Bottenstein & Sato, 1979), at 37°C in 5 or 7% CO _2_, then fed three times a week by replacing half the medium with serum-free differentiation media. All DMEM contained 100 U/ml penicillin and 100 μg/ml streptomycin. From DIV 12 onwards, feeding was done with insulin-free differentiation media. The effect of Activin-A (AA; R&D Systems, Cat. 338-AC-010; Lot BNV3313053) and recombinant human fibroblast growth factor 9 (FGF9; R&D Systems, Cat. 273-F9-025; Lot ON1413121 or ON1413041) on myelinating cultures was investigated between 15 (AA) or 18 (FGF9) and 28 DIV, on cells grown on multi-well microplates. For treating with ‘myelin debris’, coverslips were transferred from 35 mm Petri dishes the day before, into 24 well dishes in maximum 500 μl differentiation media.

### Treatment of cell grown on multi-well microplates

To minimise ‘edge-effects’ due to increased rate of evaporation or warming of media, cells were not plated in the wells on the outer edges of the microplate. Instead, these were filled with Hank’s Balanced Salts solution. DMSO (1% v/v), AA (1–100 ng ml ^-1^) or FGF9 (100 ng ml ^-1^ were added to wells following a pre-generated random pattern that varied from one experimental repeat to the next, to avoid potential effects related to the location of the wells on the microplates.

### Live imaging dishes

A protocol for custom made imaging dishes is described in the supplementary protocol document ( Supplementary File 2). Briefly, 3 x 11 mm diameter holes were burred in the bottom of 35 mm Petri-dishes (Falcon Ref. 353001) and 25 mm diameter glass coverslips (Menzel-Glaser 0,17 +/- 0,01 mm; Starke 1,5; Lot #004710182; Thermo Scientific) were stuck to the base of the dishes using non-toxic glue. Dishes are cleaned and sterilised then coated with poly-L-lysine. Live imaging dishes were alternatively purchased from MatTek Corporation, Ashland, USA or custom made ( Kline, 2009).

### Substrates for cell adhesion

Poly-L-lysine (PLL; 13.3 μg/ml) in water or boric acid buffer (50 mM boric acid, 23.5 mM sodium tetraborate, pH 8.5) was used to coat 13 mm diameter glass coverslips (Fisher Scientific, Leicestershire; 631-0150), imaging dishes, microelectrode arrays or microplates (96 [Greiner 655891] or 384-well [Greiner 781856] Sensoplate Plus, black, 175 µM glass bottom, Greiner Bio-One) for 1–12 hours at 37°C, after which the PLL was aspirated, the glass washed three times in dH _2_0 and air dried in the laminar flow hood. Empirically, we found the cultures were far less reproducible if PLL was prepared in water compared to boric acid buffer.

### Myelin isolation from mouse spinal cord

A step-by-step protocol is provided in the supplementary protocol document ( Supplementary File 3). Adult (P45-P120) wild type or *Plp1* transgenic mice were killed in gradually increasing levels of CO _2_ and the spinal column was severed at the lumbar and cervical regions. The spinal cord was rapidly removed by introducing sterile saline under pressure to the lumbar region of the spinal canal, using an 18-gauge hypodermic needle attached to a 5 ml syringe. Cords were immediately processed or snap-frozen and stored in liquid nitrogen. Myelin was harvested following the method of ( Norton & Poduslo, 1973), with slight modification. All steps were performed at 4°C, using filter sterilised solutions prepared from cell culture grade diluents or MilliQ water. Cords were homogenised in sterile 0.85 M sucrose solution in 10 mM HEPES using a polytron homogeniser at full speed for 12 strokes. Three ml of a 0.25 M sterile sucrose solution in 10 mM HEPES was slowly added on top of 7.5 ml of the homogenate. The samples were spun at 70,000 x g for 90 minutes at 4°C in a Beckman SW41 rotor. The interface between the sucrose layers, containing the membrane fractions, was gently aspirated and washed by vortexing in 6 ml chilled MilliQ water then spun at 23,000 x g for 30 minutes in a Beckman J21 rotor, to remove the excess sucrose. This osmotic shock was repeated twice more. Following a final 19,000 x g spin, the resultant myelin-enriched pellet was resuspended in cell culture grade phosphate buffered saline (PBS; Sigma-Aldrich, catalogue number 806552). The protein concentration was measured using Pierce BCA protein assay kit (Thermo Fisher Scientific, catalogue number 23225) and this myelin fraction was tested for sterility (by incubating a sample in cell culture media at 37°C for 7 days), and labeled with NHS-Rhodamine Antibody Labeling Kit (Thermo Fisher Scientific, product #53031), according to the manufacturer’s instructions.

### Addition of exogenous myelin to myelinating cell cultures

A rhodamine-labeled myelin enriched tissue fraction (2 mg protein ml ^-1^ myelin homogenate) was added to the myelinating cultures at DIV 21, DIV 25 or DIV 27 (all ± 1 day), to a final concentration of 0.075 or 0.1 mg protein ml ^-1^ (unless otherwise indicated) for respectively, 7, 3 or 1 DIV. Cell culture grade PBS alone (to the same volume as the myelin emulsion), or 3 × 10 ^6 ^one μm diameter Flurobite fluorescently labeled latex beads (Polyscience, Park scientific, Northampton, UK) were added in parallel. Two coverslips, each plated at the start with 150,000 spinal cord cells, were treated with wild type myelin, *Plp1* tg myelin, PBS or latex beads. Cells were fixed and stained with rat anti-CD45 (Serotec, catalogue number MCA 1388, monoclonal; 1 in 600) to label microglia at DIV 28 (± 1 day) for analysis. Independent myelin preparations were used for each experimental repeat.

### Antibodies and immunocytochemistry

A supplementary protocol document is attached ( Supplementary File 4). In general, cell cultures were fixed with 4% paraformaldehyde for 10–20 min at room temperature (RT), washed in PBS, permeabilized in 0.5% Triton X-100/PBS for 15 min at RT or in methanol for 10 minutes -20°C, washed with PBS, and blocked with 1% BSA/10% horse serum/PBS or with 10% goat serum/PBS for 1 hour at RT. Primary antibodies used were: rat anti-MBP (MCA4095; 1:500); rat anti-CD45 (MCA 1388; 1:300); rat anti-mouse CD68 (MCA1957T; 1:200; all Serotec Ltd, Oxford, UK), mouse anti-CNP (SMI191; 1:1000) and rat anti CD11b (Biolegend 101205 1:50; both Cambridge Bioscience, Cambridge, UK), SMI31 mouse antibody to phosphorylated neurofilament (1:1500; Affiniti Research Products Ltd., Derbyshire, UK), rabbit anti-Caspr (1:1000; kindly provided by Professor E. Peles), rabbit anti-green fluorescent protein (ab6556; 1:1000; Abcam, Cambridge, UK), rat anti-lysosome-associated membrane glycoprotein 1 (1D4B; 1:2 Developmental Studies Hybridoma Bank, Iowa, U.S.A.), mouse anti-myelin oligodendrocyte glycoprotein, (1:200, clone Z2 provided by C. Linington), rabbit anti-ionized calcium binding adapter molecule 1 (Iba1; 019-19741; 1:800, Wako, Neuss, Germany). These were diluted in blocking buffer and incubations performed at RT for 1 hour or overnight at 4°C. Anti-CD45 worked best on acetone fixed cells (10 mins, -20°C). Bound antibodies were visualised using appropriate combinations of species/isotype-specific fluorochrome-conjugated secondary antibodies (1:400 [or in later experiments, 1:1000] 488 goat-anti mouse IgG1, catalogue number A2112; 568 goat anti-mouse IgG1, catalogue number A21124; 488 goat-anti-rat IgG, catalogue number A11006; 568 goat anti-rat IgG, catalogue number A11077; 488 goat-anti rabbit IgG, catalogue number A11008; 568 goat anti-rabbit IgG, catalogue number A11036; all Alexa Fluor, Life Technologies) after incubation at RT for 15–60 minutes. Nuclei were stained with DAPI (2 µg/ml) for 5 minutes, then wells of the multi-well microplates were filled with 100 μl PBS (although Mowiol also works well, and solidifies). Coverslips were mounted on glass slides in Citifluor AF1 mounting medium (Agar Scientific, Essex, UK, catalogue number AGR1320) or Mowiol 4-88 prepared as described in the supplementary protocol document, by dissolving 2.4 g Mowiol 4-88 (#81381, Sigma-Aldrich) in 6.0 g analytical grade glycerol, 6 ml distilled water, 12 ml 0.2 M Tris pH 8.5. Plates were imaged on an IN Cell Analyzer 2000 (GE Healthcare) and coverslips were imaged using wide-field fluorescence microscopy.

### Image Acquisition in Cell Analyzer

The IN Cell Analyzer 2000 (GE Healthcare) was used to acquire multiplexed wide-field fluorescent images in 96-well microplate format with a Nikon 10X 0.45NA Plan Apo objective, with the following channels 490/20 nm & 525/36 nm (green), 579/24 nm & 624/40 nm (red) and 350/50 nm & 455/50 nm (DAPI). Using these three channels, six fields of view were imaged per well. Flat Field Correction (illumination correction) was used as an image pre-processing step during image acquisition. Axonal density (area stained with antibody SMI31, anti-neurofilament), myelin (area stained with anti-MOG or anti-MBP) and cell counts (number DAPI +ve nuclei) were quantified using CellProfiler image analysis software version 2.1.0 ( Carpenter *et al*., 2006). The pipelines developed for this study are available from GitHub. A supplementary protocol document is attached ( Supplementary File 5). Briefly, images were coded with parental metadata (imageID, wellID) and also with row/column metadata. Quality control of the images was based on cell numbers, axon density and artefacts. Nuclei were identified in the DAPI image as primary objects after image thresholding using the Otsu global method. Shape was used to segment closely spaced cell. Images with less than 1500 nuclei/image were not used for subsequent analyses. Axonal density was measured in the red image (SMI31) after global thresholding using the Otsu, three classes weighted variance method. Total area occupied by phosphorylated neurofilament was measured and image sets with less than 40% ‘axonal density’ were not used for subsequent analyses. In the green channel (MBP), after applying an Otsu global threshold, myelin-like sheaths were identified using compactness ≥ 2.5 and form factor ≤ 0.2. Nuclei count, total area, axon area and myelin area measurements were exported to a comma-delimited spreadsheet for data analysis. The average value obtained from all images from each of the treatment conditions, from a single independent cell culture, were considered one independent experimental unit, unless otherwise stated in the Figure legends.

### Wide-field fluorescence microscopy

To quantify cellular parameters in myelin treated cultures, 10 images (selected at random on the blue [DAPI] channel) each of the green (CD45), red (rhodamine-labelled myelin) and blue channels were captured using a x20 objective over two 13 mm cover slips per condition, for each independent experiment, using a CCD camera system (Photonic Science Colour Coolview) and ImagePro 6.0 software (Media Cybernatics, Silver Spring MD). To calculate CD45 +ve cell density, manual counts of CD45 positive cells containing a DAPI-labelled nucleus were made from each image within an AOI of 124384 μm ^2 ^and the sum of the values were converted to cells per mm ^2^. To quantify myelin uptake, the number of CD45 +ve cells containing rhodamine-labelled myelin per AOI was expressed as a percentage of all CD45 +ve cells per AOI. The experimenter was blinded to the ‘genotype’ of the myelin during cell quantification. Immunostained cultures were also imaged on a Zeiss Axioimager M2 wide-field microscope with Zen 2012 (Blue Edition) version 1.1.2.0 software.

### Cytokine arrays

Cytokine arrays were used to provide semi-quantitative data on cytokines and chemokines produced in response to damage and pathogen associated molecular patterns. Cultures were incubated with cell culture grade PBS, myelin of either genotype in PBS (0.1 mg myelin-protein ml ^-1 ^final concentration), or LPS (100 ng ml ^-1^; E-coli mutant O111:B4; VWR). One ml conditioned media (CM) was collected at DIV 27, spun at 12470 g for 1 minute and the upper 800 μl was stored at -80°C until required. Semi-quantitative analysis of cytokine/chemokine levels was performed using Proteome Profiler ^TM^ Array, Mouse Cytokine Array Panel A (ARY006; R&D Systems Europe Ltd., Abingdon, UK), according to manufacturer’s instructions. Briefly, nitrocellulose membranes, containing 40 anti-cytokine antibodies in duplicate, were blocked in 2 ml of blocking buffer (array buffer 6) on a rotating platform for one hour. CM from matched samples were probed in parallel. Eight hundred μl CM was added to 0.5 ml of array buffer 4 and then adjusted to 1.5 ml with array buffer 5. Fifteen µl of reconstituted Cytokine Array Panel A detection antibody cocktail, containing biotinylated antibodies, was added to each CM sample, for 1 h RT. After removal of array buffer 6, the 4 nitrocellulose membranes (1 per condition) were incubated in the sample/antibody mixture overnight at 4°C. Membranes were incubated in 1.5 ml of streptavidin-HRP (in Array Buffer 5) for 30 m at RT then treated with Pierce enhanced chemiluminescence western blotting substrate catalogue number 32106 (Perbio Science UK Ltd., Cramblington, UK) and subsequently exposed to x-ray film for between 1 and 20 minutes. X-ray films were scanned and made into digital images and the volume of each spot (minus the average edge volume) was quantified using array analysis software ( TotalLab TL100 Array v2008; Newcastle upon Tyne, UK). For each membrane, the signal volume of each of two paired spots (representing a single cytokine) was normalised to the average spot volume of 6 positive control spots on the same membrane, and the average value of the paired spots, as a percentage of the control spot value, was derived. This method corrected for different incubations and/or exposures across different independent experiments. The experimenter was not blinded during this experiment or its analysis, but the automated quantification prevented experimenter bias. Medium from each of the four conditions, from a single independent cell culture, was considered one experimental unit.

### Electrophysiology

Dissociated E13 spinal cord cells were established on MEAs (60MEA200/30iR-Ti-gr; Multi Channel Systems, Reutlingen, Germany) as described in ‘Myelinating Cell Cultures’. Electrophysiological recordings were performed from DIV 20-30. Sixteen channels were read simultaneously at 37°C in differentiation medium, in a custom-built laboratory MEA holder connected to a 16-channel amplifier (A-M Systems; Washington, USA). The array was subsequently rotated in the holder in order to read from all channels. Three minutes of extracellular recording were collected per channel (High Pass: 3 Hz; Low Pass: 500 Hz, Gain 20 k; Notch On). A semi-permeable membrane (fluorinated ethylene-propylene) was placed on top of the array, allowing cultures to be returned to the incubator and recordings to be made on subsequent days. Acquired data were analysed off-line. Modulators of neural activity were added directly to the bath: tetrodotoxin (TTX; 1 µM; Tocris Cat. 1069), cyanquixaline (CNQX; 5 µM; Tocris Cat. 1090), picrotoxin (100 µM, Sigma-Aldrich, Cat. P1675).

For single cell recording, whole-cell current clamp recording was performed using an Axopatch 200B amplifier with a Digidata 1440A digital acquisition system and pClamp 10 software. Experiments were performed at 37°C in atmospheric CO _2_ using an extracellular solution containing identical ionic concentrations to the cell culture media (in mM): 110.3 NaCl, 5.3 KCl, 1.8 CaCl _2_, 0.8 MgCl _2_, 10 HEPES, 25 glucose, pH 7.4. The pipette solution contained (in mM): 135 K-gluconate, 2 MgCl _2_, 2 Na-ATP, 0.5 Na-GTP, 10 HEPES, 0.5 EGTA, pH 7.2. Borosilicate glass pipettes were pulled to a resistance of 3-8 MΩ. TTX and other modulators (see above) were added directly to the tissue chamber.

### Live imaging of myelin phagocytosis

Spinal cord cells were plated on 35 mm diameter glass bottom Petri dishes (mentioned previously) and cultured for 20–22 days. Immediately prior to imaging, rhodamine-labelled myelin was vortexed and added to the Petri dish at final concentration 0.05 mg myelin-protein ml ^-1^. The dish was set in a Nikon TE 2000 time-lapse microscope inside a temperature/CO _2_-controlled chamber. Using Metamorph 7.5.2 imaging software set for multi-stage positions and multiple wavelengths, positions of interest were selected on the bright field (phase), cherry (rhodamine) and CFP channels, and the co-ordinates recorded. Focus was set and maintained using a PSF perfect focus control. Images were taken every 15 minutes over a 15 h period. AVI videos of the stills collected were then generated using ImageJ 1.44 software.

### Western blotting

Cell lysates from siRNA studies or spinal cord homogenates were prepared to 1 mg protein ml ^-1^ in RIPA lysis buffer system with protease inhibitors (sc24948; Insight Biotechnology), plus 5x loading buffer (Sodium dodecyl sulphate/Dithiothreitol denaturing buffer [SDS/DTT]), and heated to 65°C for ten minutes. Lysates (see Table 1 for protein amounts) were run on a 4–12% gradient NuPAGE bis-tris acrylamide gel (Invitrogen, Paisley, UK) at 200 volts for 40–50 minutes and transferred using a semi-dry system to a PVDF membrane (Millipore, Watford, UK) at 225 mA for 1 h per 2 gels. PVDF membranes were blocked in 5% milk in Tris-buffered saline/0.01% tween (TBS/T; pH 7.4) and incubated overnight with primary antibody ( Table 1) in blocking solution at 4°C, with gentle agitation. Following thorough washing, membranes were incubated in horseradish peroxidase (HRP)-conjugated goat anti-mouse or goat anti-rabbit secondary antibodies (New England Biolabs, Dundee, UK) in blocking solution ( Table 1) for 1 h RT. Following thorough washing and incubation in Pierce enhanced chemiluminescence western blotting substrate (Thermo Fisher Scientific, catalogue number 32106)), the PVDF membrane was exposed to x-ray film (AGFA) for 30 sec to 20 min to obtain optimal exposures.

**Table 1.** Antibody and lysate details for western blots.

	Supplier	Cat. No.	Host	Approx. Mol weight	1° ab	2° ab	Cell Lysate	Spinal Cord Lysate
GAPDH	Abcam	Ab9845	Rabbit	40 kDa	1 in 1k	1 in 5k	5 μg	5 μg
CNP	Cambridge Bioscience	SMI-91R500	Mouse IgG1	46 and 48 kDa	1 in 5k	1 in 2k	20 μg	5 μg
βActin	Sigma-Aldrich	A1978	Mouse IgG1	43kDa	1 in 1k	1 in 5k	5 μg	5 μg
226 (PLP /DM20)	N.P Groome		Rabbit	27 kDa	1 in 50 k	1 in 5k	20 μg	2 μg
MAG 248	N.P Groome		Rabbit	100 kDa	1 in 1k	1 in 5k	20 μg	5 μg
Mitofusin 2	Sigma-Aldrich	M6319	Rabbit	86 kDa	1 in 1k	1 in 5k	20 μg	5 μg
OPA1	GeneTex	GTX129917	Rabbit	112 kDa	1 in 1k	1 in 5k	20 μg	
NF160	Sigma-Aldrich	N2787	Mouse IgG1	160 kDa	1 in 100 k	1 in 10k	20 μg	2 μg
SMI31	Affiniti Research Products Ltd	SMI 31P	Mouse IgG1	>200 kDa	1 in 100 k	1 in 10k	20 μg	2 μg
Parkin	Santa Cruz	sc-32282	Mouse IgG2b	52 kDa	1 in 500	1 in 5k	20 μg	
Cyclophilin B	Abcam	ab178397	Rabbit	21kDa	1 in 1k	1 in 10k	5 μg	5 μg
Ac. tubulin	Sigma-Aldrich	T7451	Mouse IgG2b	55kDa	1 in 1k	1 in 10k	1 μg	5 μg
Ty. tubulin	Sigma-Aldrich	T9028	Mouse IgG3	55kDa	1 in 1k	1 in 10k	1 μg	5 μg

### siRNA

Dharmacon’s Accell siRNA passive delivery system including ‘smart pool’ siRNA (Perbio Scientific) for “difficult to transfect cells” ( Mir & Le Breton, 2008; Shen *et al*., 2008), was used. Qualitative assessment indicated that cultures incubated in Accell DM supplemented with biotin (10 ng/ml final) and D-glucose (4500 μg/ml final) (referred to as Accell DM ^+^), fared better than cells incubated in Accell DM alone, therefore siRNA was routinely added to the cultures in DM ^+^. At DIV 20, cultures were treated with (i) Accell DM ^+^ (ii) 1 μM non-targeting (NT) siRNA, (iii) 1 μM *CyB* siRNA or (iv) 1 μM *Cnp1* siRNA (all ‘smart pool’) in Accell DM ^+^ for 72 hours between DIV 20-23 and for a further 72 hours, between DIV 28-31. Between incubations with siRNA, cultures were fed as usual with differentiation media minus insulin. Cell lysate from each of the five conditions, from a single independent cell culture, was considered one experimental unit.

### Statistics

Statistical analyses were made using GraphPad Prism versions 5.0, 6.0 or 7.0 for Windows (GraphPad Prism Software, San Diego, USA). Significance levels were set to p < 0.05. One-way ANOVA using Bonferroni’s Multiple Comparison Test was used to determine effects of siRNA, comparing all conditions to NT siRNA. For analysis of myelin uptake by CD45+ve cells between genotypes and across time points, a two-way ANOVA followed by Tukey’s multiple comparisons test was used. For analysis of steady state levels of 40 cytokines on proteome arrays, ‘multiple Student’s t tests’ were used to compare between myelin genotypes or between myelin genotype and PBS. Unless otherwise indicated, each n value derives from a single independent cell culture, comprising multiple wells or coverslips, representing technical replicates of the various treatments. In general, statistically tested experiments were carried out on 3–5 independent cell cultures, usually comprising 2 or more technical repeats (as indicated in Figure Legends or text). Up to 7 cultures per protein were examined by western blotting following treatment with siRNA, due to variation in the arbitrary units’ values from experiment to experiment.

## Results

### Spinal cord-derived cell cultures recapitulate functional properties of CNS white matter

**Figure 1.**
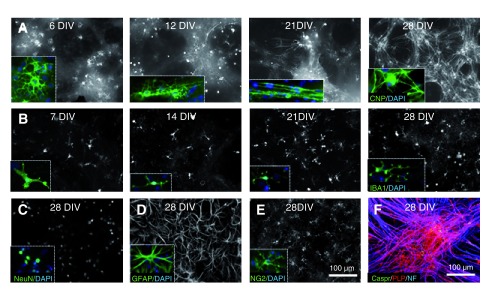
Embryonic day 13 mouse spinal cord cells differentiate over time into the major neural cell types of the nervous system. **A**. The morphology of CNP positive cells changes with time in culture. At 6 DIV, multiple cell process extend from the cell body and the cells have a ‘lacy’ appearance, as shown in the inset. By 12 DIV, a small number of myelin-like sheaths are present; often a single single sheath can be observed running in a line through one axis of an otherwise ‘lacy–appearing’ cell. In the inset, two such sheaths can be observed along 3 adjacent ‘lacy-appearing’ cells. By 21 DIV, many cells have extended multiple myelin-like sheaths and by 28 DIV few ‘lacy-appearing’ cells remain and a dense network of myelin sheaths are visible. When stained with antibodies to PLP/DM20, Caspr and phosphorylated neurofilament (NF), many axons are covered by consecutive sheaths with nodes of Ranvier straddled by Caspr positive paranodes (bottom right image). **B**. Iba1 +ve microglia are present at all stages examined. At 7DIV most microglia appear amoeboid but become ramified and extend multiple processes over time (insets). **C**. Antibody to NeuN labels neuronal nuclei and sometimes staining extends into the cytoplasm, but rarely enters the cell processes. **D**. GFAP positive astrocytes are found throughout the culture, ( **E**) as are NG2 +ve OPCs. **F**. Combined staining with antibodies to PLP/DM20, Caspr and phosphorylated neurofilament (NF), reveals that many axons are covered by consecutive sheaths with nodes of Ranvier straddled by Caspr positive paranodes. Images A–F were contrast enhanced to ease viewing.

**Figure 2.**
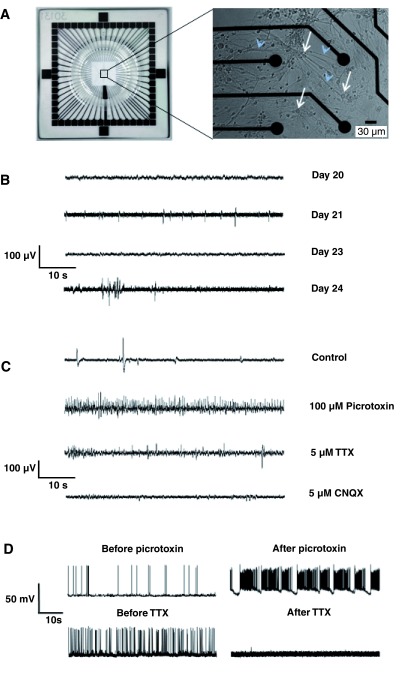
Myelinating cell cultures are spontaneously electrically active. **A**. Low power view of the whole MEA (left); all 59 recording electrodes and the ground electrode are concentrated in the center of the array. In the high-power image (right), four electrodes (black circles) can be observed in relation to the cell bodies (white arrows) and processes (including axons; blue arrowheads) of neurons and glia, on an MEA. To facilitate viewing, we selected a region in which cell density is lower than normal. Extracellular activity can be assessed from each of 59 recording electrodes. **B**. Extracellular recordings from a single electrode between 20 and 24. DIV. Each trace represents the sum of the activity detected at that electrode, which is in direct contact with multiple neuronal and glial cell bodies and processes. ‘Fast’ spike-like activity is evident at DIV 21 and ‘burst-like’ activity develops by DIV 24. **C**. Additive pharmacology on a DIV 24 culture (sequential administration of drugs without washout) demonstrates *in vivo*-like neuronal network activity of spinal cord myelinating cultures. The recording shows that addition of picrotoxin, a GABAergic inhibitor, evokes a massive increase in ‘fast’ neuronal activity (second versus first trace). Subsequent addition of TTX (a Na ^+^ channel blocker) inhibits much of the ‘fast’ spike-like (presumably axonal) activity. CNQX (a blocker of AMPA/kainate receptors) blocks excitatory synaptic activity. The remaining slow potential changes probably represent local spontaneous depolarizations (at neuronal and glial cell bodies). **D**. Examples of whole-cell current clamp recordings from single cells, presumably neurons, firing spontaneously at a resting potential of around -50 mV. Top, trace before and 5 minutes after the addition of 1 µM TTX (top). Bottom, trace from a second cell before and 5 minutes after the addition of 100 μM picrotoxin. TTX consistently blocked activity (n = 6 cells), as expected, whilst picrotoxin caused regular burst spiking in every cell tested (n = 18 cells), but the effect on overall spike rate was inconsistent, presumably reflecting the phenotype of the cell.

The function and structure of CNS white matter is dependent on a complex interplay between axons, oligodendrocytes, astrocytes and microglia; including physical, metabolic, receptor-dependent signaling and gap junction-mediated cell-to-cell communication. Myelinated cultures derived from E13 mouse spinal cord, recapitulate this cellular complexity ( Figure 1A–E), and by day *in vitro* (DIV) 24-28, comprise consecutive myelinated internodes separated by nodes of Ranvier ( Figure 1F). To define this system in terms of function, we began by examining its electrical properties using microelectrode arrays (MEAs) ( Figure 2A), each comprising 59 recording electrodes. The recordings obtained at a single electrode represent the combined extracellular responses of neurons and glia. Recordings taken daily between DIV 20 and 24, when myelination is occurring, demonstrated the gradual changes in electrical activity, starting with single isolated spikes and progressing to burst-like activity ( Figure 2B). Additive pharmacology (sequential administration of drugs without washout) demonstrated distinct ‘network-like’ aspects of the neuronal activity ( Figure 2C), not unlike the fictive bursting activity seen in isolated intact spinal cord segments ( Grillner *et al*., 1981). Picrotoxin, a GABAergic inhibitor, evoked enhanced firing (second versus first trace) and subsequent addition of TTX (a voltage-gated Na ^+^ channel blocker) blocked spike-like (presumably axonal) activity, whilst CNQX (blocker of AMPA/kainate receptors) further blocked (presumably) excitatory post-synaptic activity. The remaining slow potential changes (final trace) probably represent local spontaneous depolarizations at neuronal cell bodies and dendrites. To provide support for these conclusions we used single cell patch clamp recording. Spontaneous action potential generation was observed in 68 out of 75 cells tested; average spiking frequency being 2.13 ± 0.32 Hz (mean ± s.e.m.). The firing frequency was highly variable between cells, ranging from 0.07 to 15.27 Hz. This activity could be enhanced with 100 μM picrotoxin ( Figure 2D) or completely blocked by 1 μM TTX ( Figure 2D). In summary, spinal cord myelinating cell cultures are electrically active and activity can be manipulated bi-directionally using pharmacology.

**Figure 3.**
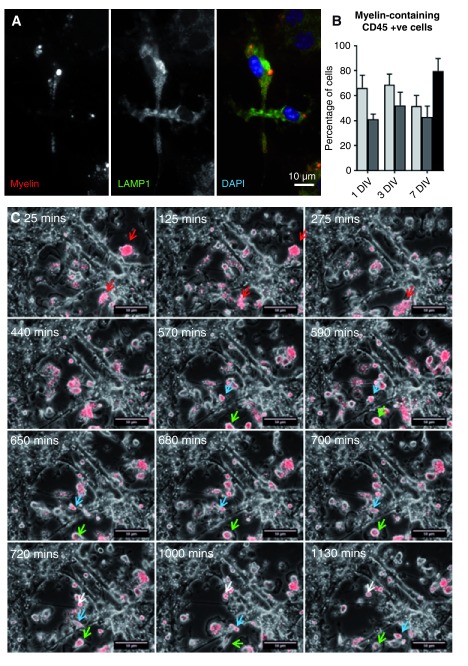
Motile microglia/macrophages take up myelin debris *in vitro*. ( **A**) ID4B antibody staining of cultures treated with rhodamine-labelled myelin debris at 27 DIV shows myelin debris (red) within lysosomal associated membrane glycoprotein 1 (green) late endosomes/lysosomes. All cells containing myelin were found to be CD45 +ve (not shown). ( **B**) After incubation with saturating amounts of myelin debris (data obtained with 0.075 or 0.1 mg myelin protein ml ^-1 ^were similar, so values from each were included providing two technical replicates) over three different time periods, the percentage of CD45 positive cells that contained ‘wild type’ (pale grey bars) or *Plp1*-tg (dark grey bars) myelin debris, which is taken up in a receptor-dependent manner, was between 40–70 % (n = 6; 2 technical replicates for each of 3 independent experiments, per time point); not significantly different from the proportion that contained latex beads (black bar), which are taken up in a receptor-independent manner (n = 3 independent experiments). Bars represent mean +/- s.e.m.. ( **C**) Time lapse stills over 1130 minutes show myelin debris (red) at time of addition (25 mins – 275mins) and then being phagocytosed by microglia/macrophages (440 mins – 1130 mins). Blue and green arrows highlight the locations of individual cells over time.

Next, we examined functional responses of microglia/macrophages, which were evident as IBA1 ( Figure 1B) or CD45 +ve cells, at all stages examined from DIV 7-28. To assess the phagocytic capability of microglia/macrophages for a relevant target, we added increasing concentrations of rhodamine-labelled myelin (subsequently referred to as ‘myelin debris’) on DIV 26 for 24 h. The proportion of CD45 +ve cells incorporating myelin reached ~66% of cells at 0.075 or 0.1 mg myelin-protein ml ^-1^. Internalized myelin appeared within lysosomal-associated membrane glycoprotein 1 +ve structures, indicating its incorporation into late endosomes/lysosomes ( Figure 3A). To assess consequences of longer-term exposure to myelin debris, we incubated cultures for 1 or 7 days with myelin (0.1 mg myelin-protein ml ^-1^) from wild type or *Plp1* transgenic mice ( *Plp1* tg line #72; Readhead *et al*., 1994); the latter is a spontaneously demyelinating model of Pelizaeus Merzbacher disease ( Anderson *et al*., 1999; Edgar *et al*., 2010) that like line #66 ( Ip *et al*., 2006) is characterized by low-level T cell infiltration into the CNS (JE unpublished observations). The proportion of CD45 +ve cells containing myelin debris did not change significantly over time, or with the ‘genotype’ of the myelin ( Figure 3B). To compare receptor-dependent versus independent phagocytosis ( Sierra *et al*., 2013), we quantified the proportion of cells incorporating myelin versus latex beads; however the proportions were similar ( Figure 3B).

In the resting brain *in vivo*, microglial soma are rather stationery but their processes are highly motile ( Davalos *et al*., 2005; Nimmerjahn *et al*., 2005). In response to focal injury, or simulation of injury by local application of ATP, cell soma ( Honda *et al*., 2001) or processes independent of the cell soma ( Davalos *et al*., 2005), converge on the injury site. To assess the dynamic behavior of phagocytic microglia/macrophages in spinal cord-derived cultures, we used live imaging immediately following the addition of rhodamine-labeled myelin debris. Cells identified as microglia/macrophages on the basis of their incorporation of myelin debris, were motile with respect to both the soma and processes, appearing to ‘gather’ myelin debris for ingestion. Qualitatively, the vast majority of exogenous myelin was internalized within the 15 h imaging period ( Figure 3C and Supplementary Video 1).

**Figure 4.**
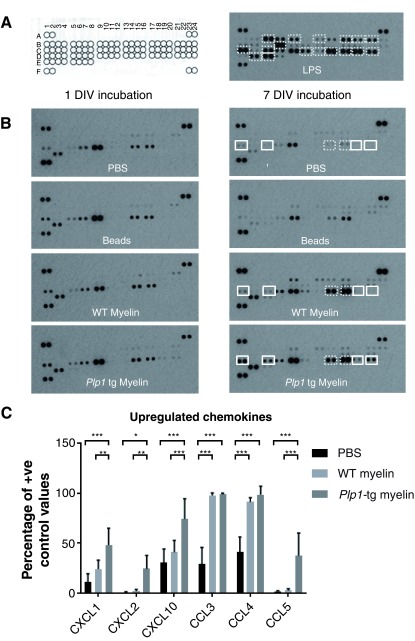
Spinal cord cultures mount appropriate responses to diverse stimuli. **A**. Map of proteome arrays shows six positive control (A1,2; A23,24; F1,2) and two negative control (F23,24) spots, alongside 40 paired cytokine-specific spots (B1,2-E7,8). The array on the right was incubated with medium from cultures treated with LPS. A number of cytokines and chemokines (highlighted with white boxes), including pro-inflammatory cytokines, were upregulated in LPS-treated cultures compared to PBS-treated controls. Quantification is provided in the text. **B**. Proteome arrays incubated with medium from cultures treated with a myelin-enriched tissue fraction from wild type or a spontaneously demyelinating model of Pelizaeus Merzbacher disease ( *Plp1* tg myelin) for 1 or 7 days. After 7 days, two chemokines were upregulated in cultures treated with wild type or *Plp1* tg myelin compared to PBS-treated cultures (highlighted with boxes with broken lines). An additional four chemokines were upregulated specifically in *Plp1* tg myelin-treated cultures compared to wild type myelin-treated cultures (highlighted with boxes with solid lines). **C**. Graph of chemokines upregulated in cultures treated with myelin-enriched tissue preparations for 7 days. Bars represent mean +/- s.e.m. n = 3 independent cultures to which 6 independent myelin preparations were added (1 preparation per independent experiment from wild type mice and 1 preparation per independent experiment from *Plp1* transgenic mice). Each cell culture (considered n = 1 independent biological repeat) comprises up to 24 × 35 mm dishes each containing 3 coverslips coated with cells. Eight 35 mm dishes are contained within one 25 mm diameter dish. Wild type or *Plp1* transgenic myelin were added to two 35 mm dishes, from within one larger 25 mm diameter dish. There was no systematic selection process for deciding which dishes would receive wild type versus *Plp1* transgenic myelin, as all dishes were identical. Raw data files attached. * p < 0.05, ** p < 0.01, *** p < 0.001.

*In vivo*, microglia not only express receptors that mediate phagocytosis of cellular debris, but also initiate responses to pathogen-associated molecular patterns (PAMPs) such as endotoxins and viral nucleic acids, and damage-associated molecular patterns (DAMPS) such as high mobility group box 1 (HMGB1) and ATP. To examine the secretory profile of spinal cord-derived cultures challenged with (i) lipopolysaccharide (LPS; from Gram-negative bacteria; 100 ng ml ^-1 ^final) or (ii) myelin debris (0.05 mg myelin-protein ml ^-1 ^final), we used a commercially available proteome array for the unbiased detection of 40 cytokines/chemokines. In preparing myelin debris, we took care to minimize the risk of contamination by endotoxin, by using cell-culture grade plastics and media wherever possible. As expected, LPS led to a significant increase in steady state levels of pro-inflammatory cytokines, TNFα, IL1-α, IL-6 (all p < 0.001), IL1β (p < 0.01); anti-inflammatory cytokine IL1-ra (p < 0.01); granulocyte colony stimulating factor (G-CSF; p < 0.001); chemokines CCL1, CCL3, CCL4, CCL5, CCL12, CXCL1, CXCL2, CXCL10 (all p < 0.001), CXCL9 (p < 0.05); soluble intracellular adhesion molecule 1 (sICAM1; p < 0.001) and TIMP Metallopeptidase Inhibitor 1 (TIMP1; p < 0.001), compared to PBS (n = 3-4 replicates from 2-3 independent cultures). A representative proteome array is shown in Figure 4A. In contrast, exposure of the cultures to myelin debris resulted in a much more restricted response, lacking upregulation of pro-inflammatory cytokines. However, chemokines CCL3 and CCL4 were significantly upregulated in cultures treated with either wild type or *Plp1* tg myelin, compared to vehicle-treated controls (PBS; Figure 4B and C), as were TIMP1 and M-CSF (p < 0.001–0.05; n = 3 independent cell cultures and 6 independent myelin preparations [3 WT and 3 *Plp1* tg]). Notably however, compared to wild type myelin debris, *Plp1* tg myelin debris was associated with significantly increased steady state levels of CXCL1, CXCL2, CXCL10 and CCL5 ( Figure 4B and C), the last two of which are strongly linked to T cell infiltration into the CNS (reviewed Hughes & Nibbs, 2018).

Together, these data demonstrate that innate immune cell properties, including phagocytosis of tissue debris and appropriate cytokine secretion profiles are reproduced in pure spinal cord-derived myelinated cultures.

### Gene expression can be knocked-down in myelinating cell cultures using siRNA

Having established these cultures recapitulate relevant *in vivo* functions, we next asked if they are amenable to manipulation using siRNA and thus capable of being used as a screen for gene silencing approaches. We selected 2 ^’^, 3 ^’^-cyclic nucleotide 3 ^’^-phosphodiesterase (CNP), which is expressed early in the oligodendroglial lineage and eventually localized to the cell soma, processes and non-compact myelin ( Trapp *et al*., 1988). We began by defining the temporal sequence of CNP expression using immunocytochemistry. We observed a small number of CNP +ve cell bodies as early as DIV 2, and by DIV 4, CNP was also observed in short cellular processes (data not provided). By DIV 6, multiple ‘lacy-appearing’ CNP +ve cells were visible ( Figure 1A) and by DIV 12, occasional myelin-like profiles were present; often as a single myelin-like sheath extending from an otherwise ‘lacy-appearing’ cell ( Figure 1A). Myelin-like profiles increased in number and extent with time, and the number of ‘lacy-appearing’ cells correspondingly diminished ( Figure 1A).

**Figure 5.**
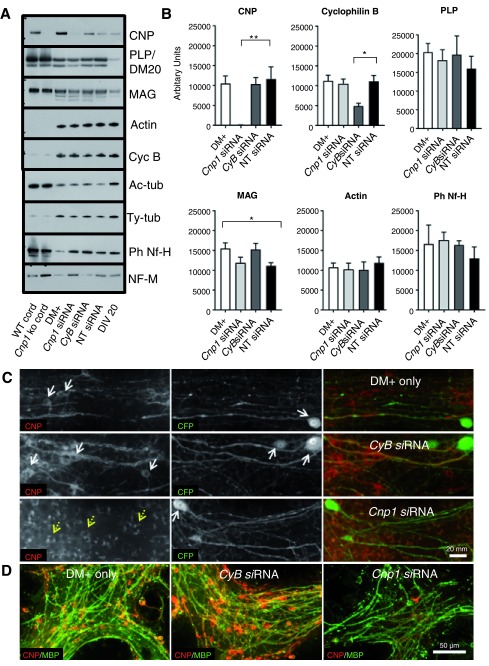
Spinal cord cultures are amenable to gene knockdown using siRNA. **A**. Western blots of (from L to R) spinal cord lysates from wild type and Cnp1 knockout (ko) mice and cell lysates from DIV 30 spinal cord cultures following treatment with supplemented incubation medium only (DM+), *Cnp1* siRNA, *CyB* siRNA or non-targeting (NT) siRNA. Lysates from time 0 (DIV 20) cultures, were run alongside. **B**. Quantification myelin proteins, housekeeping proteins and axonal proteins in siRNA treated cultures. Bars represent mean +/- s.e.m. Values were obtained from n = 4–7 independent cultures. Seven independent experiments were undertaken but some proteins were only examined in 4, 5 or 6 of these as shown in the attached raw data files. All 7 experiments were probed for CNP and 6 of 7 were probed for cyclophilin B, against which the siRNA was designed. * p < 0.05, ** p < 0.01. **C**. Micrographs of cultures prepared from Thy1-CFP mouse embryos, treated with DM+, *CyB* siRNA or *Cnp1* siRNA, and stained with anti-GFP (binds CFP which is expressed in a subset of neurons) and anti CNP. White arrows point to CNP +ve oligodendrocyte cell bodies or CFP +ve neuronal cell bodies. The punctate ‘staining’ indicated with broken yellow arrows represents autofluorescent lipofuscin granules, which are prevalent in these cultures. CNP is markedly reduced in *Cnp1* siRNA-treated cultures. **D**. Micrographs of cultures treated with DM+, *CyB* siRNA or *Cnp1* siRNA, and stained with anti-MBP (MBP is located in compact myelin) and anti-CNP (CNP is located in non-compact myelin). CNP is markedly reduced in *Cnp1* siRNA-treated cultures, but MBP appears unchanged.

To deplete *Cnp1* mRNA, cultures were incubated intermittently with siRNA from DIV 20, when CNP was already expressed ( Figure 1A and Figure 5A), until DIV 31. *CyB* siRNA was used as a positive control. By western blot, steady-state levels of CNP or cyclophilin B were significantly reduced by their respective siRNAs, compared to non-targeting (NT) siRNA-treated controls ( Figure 5A and B). In contrast, steady-state levels of proteolipid protein (PLP), myelin associated glycoprotein (MAG), actin, cyclophilin B or phosphorylated neurofilament heavy chain (Ph NF-H), were not altered in *CyB* or *Cnp1* siRNA-treated cultures compared to NT siRNA controls. With the exception of MAG, levels of all proteins were similar in NT siRNA-treated cultures compared to cultures treated only with Accell DM ^+ ^incubation medium ( Figure 5A and B). Qualitatively, neurofilament medium chain (NF-M), acetylated tubulin, tyrosinated tubulin ( Figure 5A) and mitochondrial-related proteins, OPA1, mitofusin and parkin (data not in the manuscript, but attached in raw data files, Exps N10, 12, 13, 23, 24 and 30, Dataset 1, ( Bijland *et al*., 2019)) were also not obviously changed under any treatment conditions, suggesting siRNA is not toxic to these cell cultures, even after relatively long incubation times. Using immunocytochemistry with antibodies to CNP (in non-compact myelin), GFP (to stain CFP +ve neurones from the Thy1-CFP mouse embryos) and myelin basic protein (MBP; in compact myelin), CNP specifically appeared reduced in cell bodies and myelin sheaths in *Cnp1* siRNA-treated cultures compared to DM ^+ ^only or *CyB* siRNA-treated controls ( Figure 5C and D).

In summary, this myelinating cell culture system is amenable to siRNA gene expression knockdown, in the absence of overt off-target effects.

### Myelinating cultures can be adapted to a semi-high throughput format

We have previously used this culture system to identify factors that influence myelination, however to increase its utility in this respect we asked if it could be adapted it to a format that would allow semi-high throughput screening of multiple factors, simultaneously.

Empirically, we have observed that poly-L-lysine in boric acid buffer is a highly effective substrate for cell adhesion, therefore 96-well microplates were prepared accordingly, and E13 mouse spinal cord cells were plated at a density of 75,000, 85,000 or 100,000 cells/well. Using immunocytochemisty to label myelin sheaths (anti-MBP), axons (SMI31, anti-phosphorylated NF-H) and oligodendroglia (anti-Olig 2), we found that a starting density of 100,000 cells/well led to a reproducible generation of myelinated axons after 24 DIV (data not shown). Qualitatively, plating densities of <100,000, led to lower levels of neuritic outgrowth and myelination.

**Figure 6.**
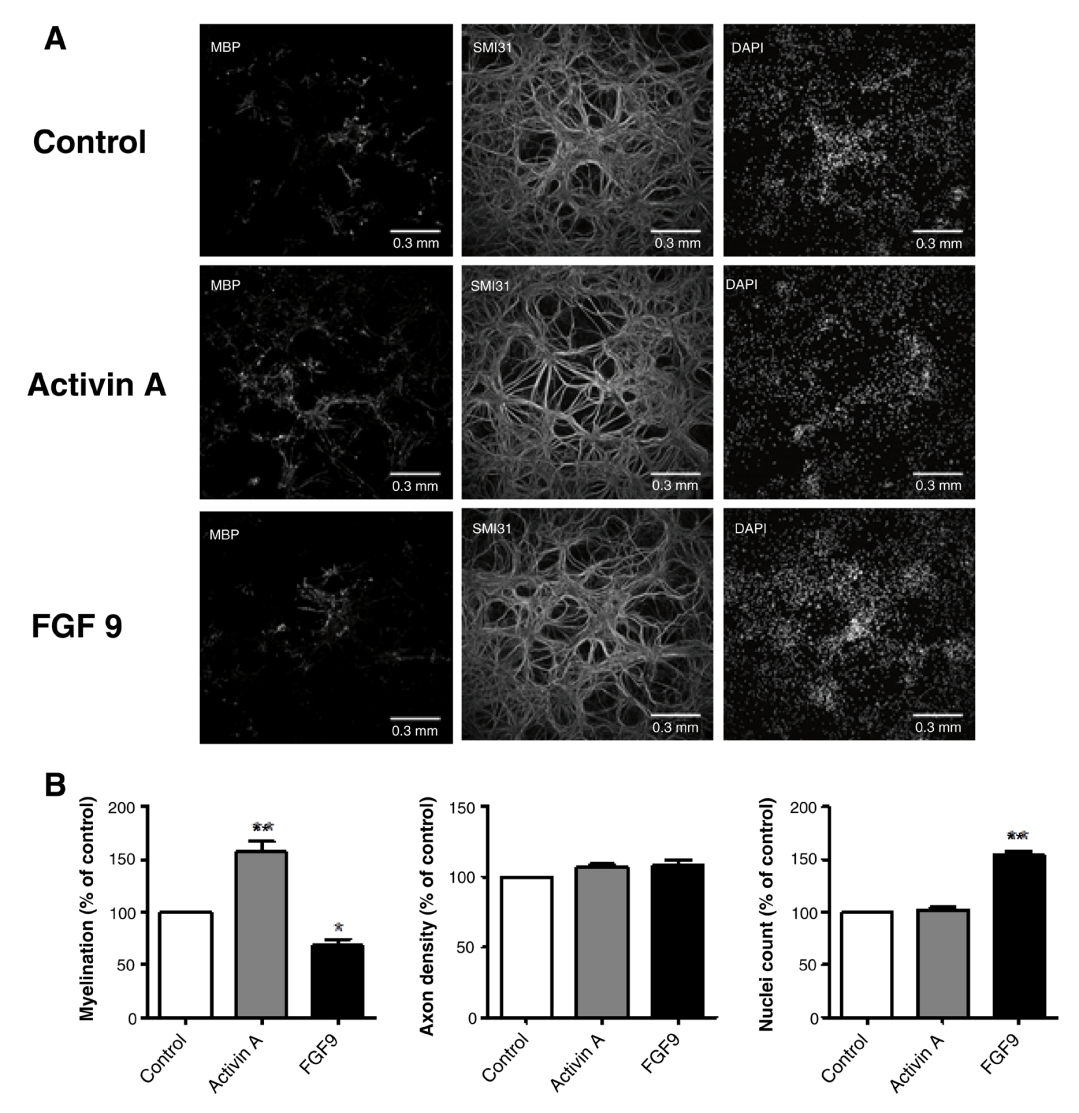
Spinal cord cultures grown in 96-well microplates are amenable to modulators of myelination. **A**. Representative immunofluorescence images of 96-well microplate myelinating cultures (28 DIV) stained for MBP, phosphorylated neurofilament (SMI31) and nuclei (DAPI). Cells were untreated or treated with Activin-A or FGF9 as described. **B**. Percentage change in myelination, axon density and nuclei count after treatment with Activin-A or FGF9. Bars represent average values from 5 independent cultures, each with 5 wells of technical replicates (wells), +/- s.e.m.. Raw data attached. Cells were allocated to different treatments as described in Materials and Methods. * p < 0.05, **p < 0.001.

**Figure 7.**
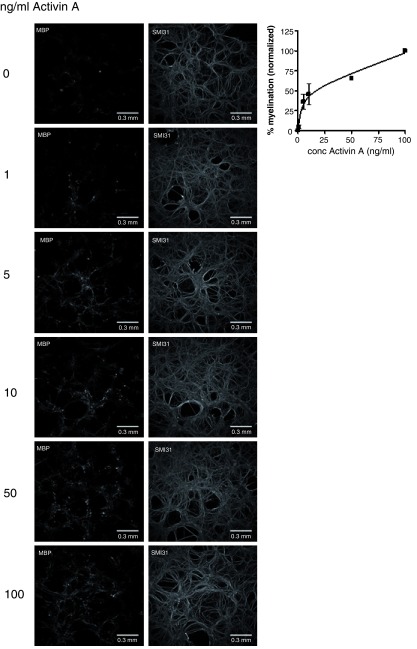
Microplate assays are sensitive to small changes in myelination. Spnal cord cultures were treated with increasing doses of Activin-A. **A**. Representative immunofluorescence images of 96-well microplate myelinating cultures (28 DIV) stained for MBP and phosphorylated neurofilament (SMI31). **B**. Dose-response curve for myelination in response to Activin-A treatment. Average values from 2 independent cultures, 5 technical repeats per culture, +/- s.e.m.

As most small molecule libraries are diluted in DMSO, we checked if DMSO adversely affected the cultures. Growing the cells in the presence of 1% DMSO had no significant effect on axon density or myelination (mean difference 2.4 % for axon density, mean difference 0.4 % for myelin, (see raw data files pertaining to Figure 6 and Figure 7, Dataset 1, ( Bijland *et al*., 2019)).

Next, we established a protocol for automated image capture using the IN Cell Analyzer 2000 High Content Screening microscope (GE Healthcare), and an automated image quantification method using CellProfiler ( Carpenter *et al*., 2006). Our analysis protocol quantified nuclear number, phosphorylated neurofilament (axonal) area and PLLS (power log-log slope, as an indicator of amount of blur in the image) and excluded images with nuclei numbers < 1500, axon area < 40% or PLLS > -2.0. For each image that passed quality control, the area occupied by myelin-like sheaths (stained with anti-MBP) and the area occupied by phosphorylated neurofilament (stained with antibody SMI31) were quantified, and MBP area expressed as a proportion of neurofilament area.

To test if our automated image capture and quantification protocols were sufficiently robust to detect changes in myelination, we treated myelinating cultures from day 19 with FGF9, a known mitogen and inhibitor of myelination ( Lindner *et al*., 2015), or from day 15 with activin A, a known enhancer of myelination ( Goebbels *et al*., 2017; Miron *et al*., 2013) until DIV 28. FGF9 induced a significant (-31.7%, p < 0.05) decrease in myelination while activin A (100 ng ml ^-1^) produced a significant (+56.5 %, p < 0.001) increase in myelination, relative to vehicle-treated cultures ( Figure 6). Furthermore, FGF9 (100 ng ml ^-1^) led to a significant (+53,8 %, p < 0.001) increase in nuclear density (average of 5 independent experiments; each with 5 technical repeats), as expected for a pro-proliferative factor ( Lindner *et al*., 2015). To test the sensitivity of our assay, we treated cultures with 1, 5, 10, 50 or 100 ng ml ^-1^ activin A and found that increasing concentrations (1-100 ng/ml) of activin A led to a dose-dependent increase in the degree of myelination ( Figure 7; average of 2 independent experiments; each with 5 technical repeats).

Thus, the methods are robust and sufficiently sensitive to detect small changes in myelination and cell densities.

As libraries of small molecules are costly, we proceeded to test whether myelinating cultures could be grown in 384-well microplates, which would allow markedly smaller amounts of compounds to be used in each assay. We plated spinal cord cells at densities of 30,000, 40,000 or 50,000 cells/well and stained the cultures with antibodies to phosphorylated neurofilament and MBP. Qualitatively, we found that the optimal plating density was 50,000 cells/well but the cultures tended to be less reproducible in this format.

An *in vitro* model for studying CNS white matter: functional properties and experimental approaches Click here for additional data file. Copyright: © 2019 Bijland S et al. 2019 Data associated with the article are available under the terms of the Creative Commons Zero "No rights reserved" data waiver (CC0 1.0 Public domain dedication).

## Discussion

Here we demonstrated that a murine, pure spinal cord-derived cell culture system ( Thomson *et al*., 2006; Thomson *et al*., 2008) recapitulates several *in vivo* cellular functions, namely, spontaneous spinal-like electrical activity; phagocytosis of tissue debris; and appropriate secretory responses to diverse stimuli. Further, the system, which is straightforward to establish, can be used to assay gene-silencing approaches; adapted to a 96-well format for semi-high throughput assays of multiple factors simultaneously; or for growth on MEAs to monitor electrical properties. These advances increase the utility of this system for studies to address white matter development, function and pathology, providing the research community with a robust experimental tool that reduces costs and minimises the use of experimental animals. In particular, the multi-well format allows large numbers of factors to be assayed in parallel, whilst simultaneously reducing the numbers of mice required to provide tissue.

Whilst this *in vitro* model of CNS white matter is not intended to replace all animal work, it can inform, or augment such experiments. For example, we previously identified pro-myelinating factors from a list of candidates generated from transcriptomic analyses of laser-captured CNS tissue from *Pten* conditional knockout and control mice, using 11 independent cell cultures (Supplementary Figure 8 h; Goebbels *et al*., 2017) from 11 pregnant dams. A similar *in vivo* experiment would first require invasive surgery to administer a demyelinating agent into the CNS, followed by subsequent administration of the pro-myelinating compounds by the same route. Given that 8 compounds were analysed ( Goebbels *et al*., 2017), a similar *in vivo* study would require the same 8 compounds to be administered to a least 11 mice per treatment group, assuming variation similar to that observed in cell culture; requiring at least 88 experimental animals. In this instance, our *in vitro* model affords an 87.5% reduction in the number of animals required compared to a similar *in vivo* experiment.

### Microelectrode arrays increase the utility of spinal cord cultures

In CNS white matter, a supra-threshold axon caliber is necessary for initiation of myelination ( Mayoral *et al*., 2018) and, when experimentally induced, is associated with *de novo* myelination of axons that ordinarily, are not myelinated ( Goebbels *et al*., 2017). Myelin formation is further modulated by neuronal signals, including activity-dependent signals ( Gibson *et al*., 2014; Hines *et al*., 2015; Mensch *et al*., 2015; Mitew *et al*., 2018). In neurons themselves, action potential firing patterns modulate the abundance of neuronal mRNAs across many functional gene categories affecting, for example, neurite outgrowth, synaptic connections and neural network formation ( Lee *et al*., 2017), whilst conversely, protein levels can modulate neuronal activity ( Lopez de Armentia *et al*., 2007). Thus, the circular relationship between fast electrical responses and longer-term changes in gene expression is important for nervous system development and plasticity ( Lee *et al*., 2017). Here we demonstrated that spinal cord-derived myelinating cultures are spontaneously electrically active, and likely subject to similar bidirectional regulation of gene expression in neurons and in oligodendrocytes.

Neural electrical activity readouts provide information on factors such as cell phenotype, metabolic status and health. As few laboratories are equipped with the necessary equipment and expertise to perform intracellular recordings, we reasoned that commercial MEAs might provide an accessible method for assaying global extracellular electrical responses. Using MEAs we identified fast spike-like, low amplitude extracellular activity that increased in frequency in the presence of the GABA receptor antagonist, picrotoxin, suggesting that inhibitory neurons suppress this spontaneous activity. Conversely, spike-like activity was reduced by the voltage-gated sodium channel blocker, TTX, indicating it represents axonal action potentials. The AMPA receptor antagonist, CNQX, blocked some of the TTX-resistant activity, suggesting it represents changes at dendrites brought about by the synaptic release of glutamate. This is compatible with ultrastructural observations in these cultures ( Figures 6F and G; Thomson *et al*., 2008). To validate these MEA data, we used single cell current clamp recording which confirmed that high frequency action potential activity was present and could be enhanced by picrotoxin, or blocked by TTX ( Figure 2) or CNQX (raw data provided separately, Dataset 1, ( Bijland *et al*., 2019)); the last demonstrating it is glutamate-dependent. However, it is important to bear in mind that the extracellular activity recorded on the MEAs represents the sum of all neuronal and glial electrical responses at the recording electrode. 

In terms of ease of use, spinal cord cells adhered well to MEAs and appeared, under phase contrast microscopy, indistinguishable from cells grown on glass. MEAs do not require the same specialist expertise or equipment needed for intracellular recordings and have the advantage that electrical activity can be assessed sequentially over days or even weeks. Further, they provide an overview of activity that cannot be achieved easily using intracellular recordings. Consequently, MEAs increase the utility of this multi-cell type culture for studies such as ion channel screening, drug testing and safety pharmacology.

### Spinal cord-derived cultures mount appropriate responses to diverse stimuli

In response to infectious, autoimmune or physical insults, microglia mount neuroinflammatory responses to combat pathogens and enhance repair and restoration of function (reviewed in Song & Colonna, 2018b; Song & Colonna, 2018a). However, this response must be finely balanced as excessive or chronic neuroinflammation can, in some circumstances, contribute to disease pathogenesis ( Song & Colonna, 2018b; Song & Colonna, 2018a; Song *et al*., 2017). Pattern recognition receptors (PRRs) on microglia play a major role in the initiation of these responses. Originally discovered for their role in recognizing pathogen-derived molecular signatures, PRRs also respond to damage associated molecular patterns (DAMPs) released by dying or damaged cells, such as ATP, high mobility group box 1 protein (HMGB1), beta amyloid and α-synuclein ( Kigerl *et al*., 2014). Chronic generation of DAMPs within the CNS can therefore run the risk of causing secondary, collateral tissue damage by sustaining long-term activation of pro-inflammatory responses (reviewed in Song *et al*., 2017).

To determine if spinal cord-derived cultures respond appropriately to a classical PAMP, we challenged them with bacterial lipopolysaccharide (LPS) and compared their response to that induced by myelin debris; sterile clearance of which is essential to promote lesion repair in demyelinating diseases such as MS and experimental models ( Kotter *et al*., 2005; Napoli & Neumann, 2009; Neumann *et al*., 2009) in a CX _3_CR1-and TREM2-dependent manner ( Cantoni *et al*., 2015; Lampron *et al*., 2015; Poliani *et al*., 2015). LPS is a well-characterized PAMP that activates toll-like receptor 4 (TLR4) to up-regulate expression of pro-inflammatory cytokines and chemokines by a wide variety of immune cells ( Chow *et al*., 1999).

As the resident immunocompetent population of the CNS, microglia express multiple toll-like receptors, including TLR4 ( Bsibsi *et al*., 2002; Zhang *et al*., 2014) and initiate inflammation in response to PAMPs by secreting pro-inflammatory factors. These include cytokines such as tumor necrosis factor (TNF)-α, interleukin (IL)-1β, and IL-6 and free radicals such as nitric oxide (NO) ( Hanisch & Kettenmann, 2007; Hines *et al*., 2013; Tambuyzer *et al*., 2009). This was replicated in spinal cord myelinated cultures, in which LPS increased secretion of pro-inflammatory cytokines (IL-1α, Il1β, IL-6 and TNFα, as well as CCL and CXCL chemokines, mimicking the CNS response induced by viruses, bacteria or parasites ( Forrester *et al*., 2018; Lobo-Silva *et al*., 2016). In striking contrast, clearance of ‘wild type’ myelin debris in these cultures did not induce an overt pro-inflammatory response, but selectively increased secretion of CCL and CXCL chemokines. This is in agreement with the homeostatic roles of microglia (and astrocytes) associated with clearance of myelin and other cellular debris injury ( Perry *et al*., 1985). 

Conspicuously however, challenging cultures with *Plp1*-tg myelin resulted in additional up regulation of CCL5 and CXCL10. Whilst chemokines facilitate context-dependent migration of all immune cells, CCL5 and CXCL10 and their receptors are particularly relevant with respect to T cell recruitment into the brain ( Hughes & Nibbs, 2018). These observations are especially interesting in light of the fact that increased numbers of T lymphocytes are present in the CNS of spontaneously demyelinating *Plp1* tg mouse models of Pelizaeus Merzbacher disease (PMD), caused by gene duplication (JE, unpublished observations and Ip *et al*., 2006). CXCL10 ** expression is induced by type I interferons to recruit T lymphocytes into the CNS to protect against neurotropic viral infections ( Liu *et al*., 2000) and it and its receptor, CXCR3, are necessary for induction of experimental cerebral malaria (ECM); probably due to the critical role CXCL10 plays in T cell-endothelial cell adhesion, and injury of the brain endothelium ( Sorensen *et al*., 2018). Further, the increased susceptibility to encephalitic symptoms after infection with West Nile virus, of individuals harbouring mutated *CCR5*, which encode the receptor for CCL5, is thought to be due to failure of trafficking of T cells into the brain ( Glass *et al*., 2005) and reviewed in ( Hughes & Nibbs, 2018). The molecular basis for the difference in chemokine induction between the two myelin ‘genotypes’ is not known; although our working hypothesis is that myelin isolated from *Plp1* tg ** mice is subtly altered allowing it to cross-link or activate additional receptors at the microglial surface ( Lennartz & Drake, 2018).

The importance of replicating the multi-cell type environment of the CNS when studying microglial functions such as these is highlighted by the fact that microglial phenotypes are modulated by neighboring cells and vice versa. For example, LPS-induced secretion of IL-1β and TNF by microglia can result in potent induction of pro-inflammatory gene expression by astrocytes *in vitro* (reviewed in Saijo & Glass, 2011), whilst signaling induced by microglial CD172, CD200R and CD45 interacting with CD47, CD200 and CD22, respectively at the neuronal cell surface, inhibits microglial activation (reviewed in Saijo & Glass, 2011).

### Spinal cord cultures can be used to test gene-silencing strategies

Neurodegenerative diseases, including those that predominantly affect white matter, such as the leukodystrophies and amyotrophic lateral sclerosis (ALS) remain largely untreatable or relatively refractory to currently available therapies. In both cases, a dominant gain-of-function effect of the protein products of mutated genes contribute to pathogenesis in some forms of these diseases, for example in familial amyotrophic lateral sclerosis (fALS) due to mutation in *SOD1* gene and in Pelizaeus Merzbacher disease (PMD) due to point mutation or duplication of the *PLP1* gene (reviewed in van Zundert & Brown Jr., 2017; Nave & Griffiths, 2004). Thus, gene silencing using siRNA or antisense oligonucleotides represents a rational approach to treatment that is potentially devoid of some of the problems caused by the broad modes of actions of small molecule-based drugs ( Zheng *et al*., 2018). A number of issues related to the systemic administration of siRNA or antisense oligonucleotides for CNS disorders remain to be overcome, such as delivery in the blood circulation, passage across the blood-brain barrier, and targeting to the appropriate cell type ( Zheng *et al*., 2018). Nonetheless, it is important to have relevant *in vitro* models to carry out preliminary tests of the efficiency of gene knockdown and to screen for off-target effects before proceeding to *in vivo* studies. This is particularly important for genes expressed in post-myelination oligodendrocytes; cells whose phenotype is altered when they wrap axons.

### Spinal cord cultures are amenable to use in semi-high throughput assays

Unlike PMD and fALS, which are genetically determined, MS is an acquired inflammatory demyelinating disease, affecting more than 2 million people worldwide ( World Health Organization, 2008). MS involves a neurodegenerative process driven in part by the failure of MS lesions to remyelinate ( Franklin & Ffrench-Constant, 2008; Franklin *et al*., 2012; Trapp & Nave, 2008). Consequently, a potential therapeutic approach is to stimulate remyelination; an effect that enhances functional recovery ( Duncan *et al*., 2009) and is predicted to reduce ongoing axonal loss ( Irvine & Blakemore, 2008; Mei *et al*., 2016). Spontaneous remyelination in MS is incomplete, leaving many axons chronically devoid of myelin ( Patani *et al*., 2007). This is thought to render them vulnerable to inflammatory insult and devoid of oligodendroglial-mediated support ( Franklin *et al*., 2012; Nave & Trapp, 2008; Trapp & Nave, 2008). In the adult brain, new myelin sheaths are formed by OPCs ( Crawford *et al*., 2016; Keirstead & Blakemore, 1997; Zawadzka *et al*., 2010), which are often abundant in MS lesions but fail to myelinate the naked axons ( Chang *et al*., 2000; Chang *et al*., 2002; Scolding *et al*., 1998). Recent studies are beginning to identify drugs, small molecules and pathways that can be modulated to overcome remyelination failure. These include Wnt/β-catenin ( Fancy *et al*., 2009), retinoid X receptor gamma (RXRγ) signalling ( Huang *et al*., 2011), muscarinic receptor antagonists such as benztropine and clemastine ( Deshmukh *et al*., 2013; Mei *et al*., 2014) activin A ( Goebbels *et al*., 2017; Miron *et al*., 2013) and drugs including miconazole and clobetasol ( Najm *et al*., 2015). However, there is still a need for a cost-effective screening strategy to identify molecules with therapeutic potential for the treatment of MS and other multifactorial neurodegenerative disorders.

A valuable pre- *in vivo* screen of candidate therapeutics should fulfil certain criteria. First, it should contain all major cell types of the CNS, to resolve off-target effects of the test agent. Second, it should be simple to establish, reproducible, sensitive and unbiased. Third, it should be unaffected by carriers such as DMSO. Fourth, it should be capable of screening a substantial number of agents while using the minimum number of experimental animals. Finally, it will provide added-value if it can be generated from mouse models of disease and/or transgenic reporter mice for live imaging or expression analysis. Here we demonstrated murine spinal cord-derived cultures meet these criteria for semi-high throughput screens and recapitulate relevant functional properties of CNS white matter. Given the ease with which they can be established, this system provides laboratories with a simple, functional and relatively inexpensive method to explore normal and pathological processes relevant to white matter in general, and spinal cord in particular.

## Data availability

The data referenced by this article are under copyright with the following copyright statement: Copyright: ï¿½ 2019 Bijland S et al.

Data associated with the article are available under the terms of the Creative Commons Zero "No rights reserved" data waiver (CC0 1.0 Public domain dedication). 

Underlying data is available from F1000Research

F1000Research: Dataset 1. An *in vitro* model for studying CNS white matter: functional properties and experimental approaches, https://doi.org/10.5256/f1000research.16802.d233269 ( Bijland *et al*., 2019)

Data is available as a zip file contain the corresponding data for the following figures:

Figure 1- Raw microscope image files

Figure 2B, C and D and for CNQX treated cell (not shown in Figure 2) – electrophysiology recording values.

Figure 3B – images and quantification of densities of CD45 +ve cells and the proportions that contain wild type, *Plp1* transgenic myelin or latex beads.

Figure 4C – scans of X-ray films of proteome arrays following treatment for 7 days with PBS, latex beads, wild type myelin or *Plp1* tg myelin, plus raw values in arbitrary units and normalised values (to positive control spots) for spot intensity for each of the cytokines assayed, plus a template for cytokine array.

Figure 5 - scans of X-ray films and raw arbitrary unit values of band sizes and intensities.

Figure 6 and 7 – images from IN Cell Analyzer 2000 and CellProfiler values for myelin, axons and DAPI +ve nuclei.

Due the size of files for the microplate images these can not be provided but are available at request from the corresponding author (JE) julia.edgar@glasgow.ac.uk

## Software availability

Analysis pipelines are available from Github: https://github.com/muecs/cp/tree/v1.0

An archived version available of the pipelines are available from Zenodo: http://doi.org/10.5281/zenodo.2533339 ( Mücklisch & Mücklisch, 2019)

Available under a 'Creative Commons Attribution 3.0 Unported License'

## Supplementary material

Detailed protocols are provided in the following Supplementary Files:

Supplementary File 1. Preparing mouse spinal cord cultures

Supplementary File 2. Preparing custom-made dishes for live imaging

Supplementary File 3. Preparing and labelling a myelin-enriched spinal cord fraction

Supplementary File 4. Immunostaining myelinating cell cultures

Supplementary File 5. Using CellProfiler analysis software

Supplementary Video 1: Live imaging of myelin phagocytosis
