# Supplementary material for: An in vitro model for studying CNS white matter: functional properties and experimental approaches
Source: F1000Res. 2019 Jan 29;8:117. [Version 1] doi: 10.12688/f1000research.16802.1 (PMC6489523; doi:10.12688/f1000research.16802.1)
Supplement: Supplementary file 4 [file f1000research-8-18368-s0003.tgz › 61fd993a-a9dd-4c7f-b0d9-bed7b47911b3_Suplementary_material_3_Myelin_preparation_and_labelling.docx]

**Preparation and labelling of a myelin-enriched spinal cord tissue fraction.**

Isolate sterile myelin from spinal cords of adult (P45-P120) wild type and homozygous *P1p-*transgenic mice using a technique based on the method of Norton and Poduslo, J Neurochem (1973) and adapted by authors MM and JME. See also Yool et al., J Neurosci Res (2001).

1. Rapidly remove the spinal cord from the spinal enclosure by introducing sterile saline under pressure using an 18 gauge hypodermic needle.
   1. Kill the mouse in increasing levels of CO_2_
   2. Cut the head off where the skull joins the spinal column.
   3. Holding the mouse in the thoracic region, cut the spinal column at the pelvis (lumbar region).
   4. The hind limbs will drop and expose the cut spinal column. Lifting this way keeps blood away from the column so you can see the column.
   5. Insert a 20 G needle attached to a saline-filled sterile 5 ml syringe, into the spinal cord at the lumbar end. The 20 G needle should wedge tightly into the spinal column. This is critical.
   6. Press hard on the syringe plunger and the spinal cord will be expelled through the cervical column.
   7. Snap free in liquid nitrogen in a cryo-tube or process immediately
   8. The whole procedure should take less than 60 seconds
2. Homogenise the cords in a 0.85M sucrose solution in 10mM HEPES, pH 7.4, using an Ultra‐Turrax T8 blender (IKA Labortechnick; IKA‐Works Inc., Wilmington, NC)  at full speed for 12 strokes.
3. Transfer 7.5ml of the homogenised tissue to Beckman tube
4. Slowly pipette 3ml of 0.25M sucrose in 10mM HEPES on top until two obvious phases can be seen.
5. Spin the samples at 70,000*g* for 90 minutes at 4˚C in an SW41 rotor in a Beckman ultracentrifuge
6. Carefully remove the upper sucrose phase with a glass pipette leaving the interface containing the membrane fraction.
7. Gently aspirate the membrane fraction and transfer to a fresh Beckman tube.
8. Osmotically shock the membrane fraction using 6 ml chilled MilliQ water (Millipore System). Vortex then spin at 23,000*g* for 30 minutes in a J21 rotor.
9. Repeat this osmotic shock process three times removing all water after each spin.
10. The final spin is performed at 17000*g*.
11. Re-suspend the myelin pellet in sterile phosphate buffered saline (PBS) without protein or phosphatase inhibitors because the myelin is to be added to cell cultures. The volume depends on the size of the pellet, but ~400 μl is a useful estimate.
12. Assay protein concentration using the Pierce BCA assay according to the manufacturer’s instructions and prepare to 2 mg protein ml^-1^ in PBS.
13. In order to visualise the phagocytosed myelin, label the purified myelin with Pierce NHS-Rhodamine Antibody Labeling Kit (Pierce, Northumberland, UK).
14. Add 40 μl of borate buffer (0.67 M) to 0.5 ml of myelin in PBS at a concentration of 2mg protein ml^-1^ to give a final concentration of borate buffer of 0.05M.
15. Add this mix directly to a vial of NHS-Rhodamine reagent, pipetting up ~10 times till all the dye is dissolved.
16. Briefly centrifuge the sample on a benchtop centrifuge to collect the sample at the bottom of the tube and incubate at room temperature for 1 hour, protected from light.
17. While the myelin is incubating, prepare the purification resin. Add 400μl of re-suspended purification resin to two separate spin columns in separate micro-centrifugation collection tubes.
18. Centrifuge the columns for 30-45 seconds at 1000 x g to remove the storage solution from the resin.
19. Transfer the spin columns containing the resin to fresh micro-centrifugation tubes.
20. After incubation (as in 16), add 250μl of the labelled myelin to each column and centrifuge at 1000 x g for 30-45 seconds.
21. Combine the samples containing the fluorescently labelled myelin from both micro-centrifugation tubes. Store the labelled myelin at -80 ºC until required.
