# Supplementary material for: An in vitro model for studying CNS white matter: functional properties and experimental approaches
Source: F1000Res. 2019 Jan 29;8:117. [Version 1] doi: 10.12688/f1000research.16802.1 (PMC6489523; doi:10.12688/f1000research.16802.1)
Supplement: Supplementary file 5 [file f1000research-8-18368-s0004.tgz › 9fb718dd-1523-4851-bdc9-e7a8e56d8384_Suplementary_material_4_Staining_myelinating_cultures_.docx]

**IMMUNOCYTOCHEMISTRY**

**Immunostaining myelinating cultures**


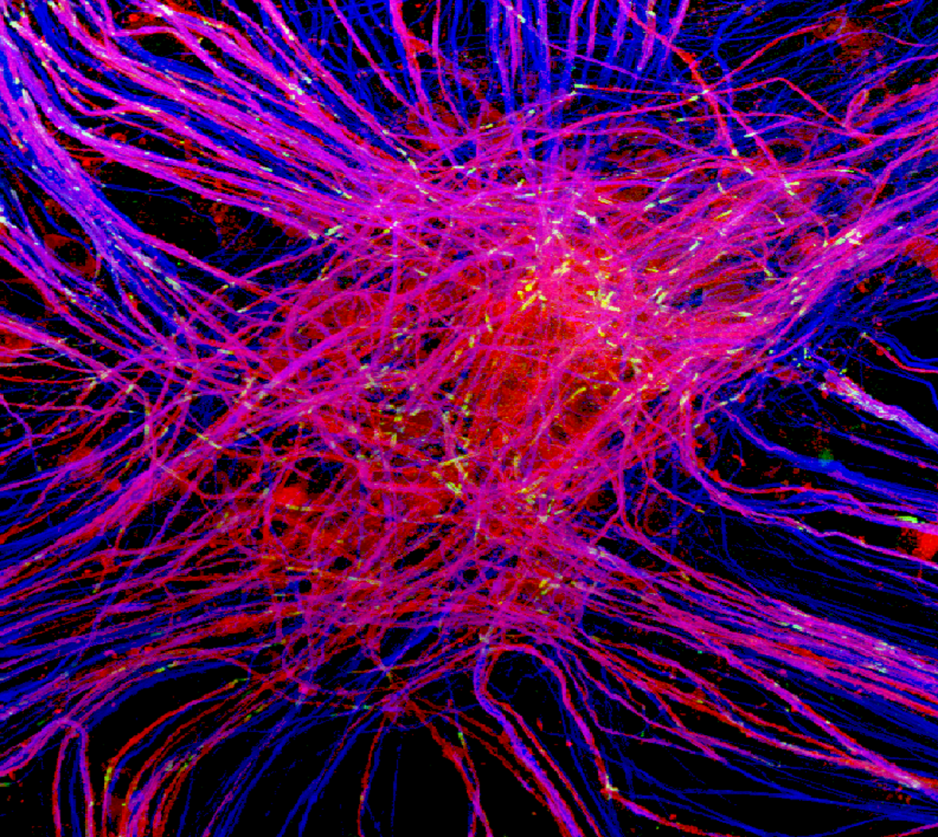


**axon/myelin/paranode**

**Figure 1. E13 mouse spinal cord cells differentiate into neurons and myelinating oligodendrocytes, *in vitro*.** Cultures were fixed and stained on day 24 *in vitro*. Myelin (red) was stained with an antibody to myelin basic protein; axons (blue) were stained with an antibody to neurofilament; and the paranodes (green) were stained with an antibody to CASPR.

To visualise **axons**, **astrocytes**, **myelin** and **microglia** we use primary antibodies directed against cell-type specific antigens, and fluorescently tagged secondary antibodies directed against one or other of the primary antibodies. Specifically, this protocol describes staining with:

1. antibodies to (i) neurofilament (NF), which labels **axons**, and (ii) glial fibrillary acidic protein (GFAP), which labels **astrocytes**;
2. antibodies to (i) NF and (ii) ionized calcium binding adaptor molecule (IBA1) which labels **microglia**;
3. antibodies to (i) NF and (ii) myelin basic protein (MBP), which labels the **myelin** **sheath**.

The anti-NF antibody was raised in mouse; the anti-GFAP and anti-IBA1 antibodies were raised in rabbit; the anti-MBP antibody was raised in rat. This information is needed in order to select the appropriate secondary antibodies.

**Overview**

1. **Fix cells** with paraformaldehyde to preserve them by cross-linking proteins
2. **Permeabilise** cells to allow antibodies to access their intracellular antigens.
3. **Block sites** where secondary antibody might bind non-specifically
4. Apply **primary antibody** to the antigen of interest
5. Apply a **fluorescently labelled secondary antibody** (green or red) to bind to the primary antibody
6. Mount coverslips onto glass slides in mounting medium with DAPI

**Materials**

- Staining tray (e.g. Corning square bioassay dish; 500 cm^2^; Sigma CLS431111-16EA)
- Parafilm laboratory film
- Fine, curved forceps
- 1.5 ml eppendorf tubes plus a vortex for mixing antibodies
- Three 500 ml bottle caps (PBS baths)
- Phosphate buffered saline (PBS) pH 7.4
- Freshly prepared 0.5% Triton X in PBS (a detergent that ‘punches’ holes in the cells to allow the antibody inside the cell)
- Blocking buffer (to block non-specific binding of secondary antibody), made with serum from the species in which the secondary antibody is raised e.g.10% goat serum in PBS
- Glass slides with frosted edge for writing experimental details on
- 13 mm diameter glass coverslips with myelinating cultures, face up in PBS following fixation in 4% paraformaldehyde for 10 mins, in 35 mm Petri dishes.
- Primary antibodies (stored frozen in small aliquots in PBS/0.01% goat serum diluted 1 in 10)
- Secondary antibodies (stored at 4 ^o^C in the dark)
- Mounting medium* containing DAPI to label cell nuclei
- Clear nail enamel to seal the coverslips to the slides if a non-setting mounting medium is used
- Positive and negative controls if testing a new antibody/new sample. When testing an antibody for the first time, always do two/three single stains (e.g. GFAP or MBP or NeuN) as well as double/triple labelling (e.g. GFAP + MBP + NeuN).

***To make Mowiol 4-88 mounting medium**

6.0 g analytical grade glycerol

2.4 g Mowiol 4-88 (Calbiochem # 475904)

6 ml distilled water

12 ml 0.2 M Tris pH 8.5

Put glycerol in 50 ml plastic conical tube. Add Mowiol and shake thoroughly. Add distilled water and leave for 2 h at room temperature. Add Tris and incubate at 50oC until the Mowiol has dissolved completely. Clarify by centrifucation at ~ 400 rpm for 20 min. Aliquot supernatant into 1.5 Eppendorf tubes and store at -20oC. If required, you can add DABCO (Diazabicyclo(2.2.2) octane, Sigma D-2522) anti-fade reagent to Mowiol, according to manufacturer’s instructions. We do not find it is required.

Protocol from Mary Osborn, in Immunofluorescence Microscopy of Cultured Cells, in Cell Biology: a laboratory handbook, Vol 2, published by Elservier

**To label cell nuclei, DAPI (2-(4-Amidinophenyl)-6-indolecarbamidine dihydrochloride, 4′,6-Diamidino-2-phenylindole dihydrochloride;** 10 mg/mL in H_2_O; Invitrogen D1306**) can be added to mounting medium to a final concentration of 2 μg/ml).**

**Protocol for staining myelinating cultures**

Practise handling coverslips with fine curved forceps before you start immunostaining. It is easy to drop them or break them.

1. Fill the 3 bottle caps with PBS (PBS baths) and cover the base of the staining tray with Parafilm laboratory film (ensure it is flat and adheres lightly to the plastic)
2. Label the lid and base of the staining tray to indicate which the ‘top’ edge is. Label the lid of the staining tray to indicate which stains you will use e.g.:
   1. NF & GFAP
   2. NF & IBA1
   3. NF & MBP
   4. NF & GFAP –ve control
   5. NF & IBA1–ve control
   6. NF & MBP –ve control
3. Place 6 x 50 μl drops of 0.5% Triton X on the left side of the staining tray, approx. 2 cm apart, in a column; each drop aligned with one of the labels on the lid
4. Carefully lift one coverslip, which is face up, from the 35 mm Petri dish and place it face down on the 1st Triton X drop. Repeat with a second coverslip and place it on the 2^nd^ Triton X drop etc
5. Incubate for 10 minutes to permeabilise the cells to provide access for the antibodies to **intracellular** antigens.
6. Meantime place 6 x 50 μl drops of blocking buffer on the staining tray, in parallel and to the right of the Triton X drops
7. Carefully lift the 1^st^ coverslip by gripping it gently with curved forceps (REMEMBER WHICH SIDE IS FACE UP AND TAKE CARE TO AVOID DROPPING THE COVERSLIP) and wash it in PBS by immersing it in each of the three PBS baths. It is important to drain onto tissue baths. Place it face down on the first of the blocking buffer drops. Repeat with remaining coverslips. Incubate for 30 minutes at room temp (RT).
8. Prepare the primary antibodies in blocking buffer (if they are already diluted for storage [recommended] take this dilution factor into account:

e.g.

dilute mouse anti-NF 1 in 1000 and rabbit anti-GFAP 1 in 1000 (i.e. 1 μl of each in 1000 μl blocking buffer);

dilute mouse anti-NF 1 in 1000 and rabbit anti-IBA1 1 in 200;

dilute mouse anti-NF 1 in 1000 and rat anti-MBP 1 in 400.

1. Clean the Triton X drops off the left side of the staining tray using tissue and replace with 1 x 40 μl drop of each primary antibody mixture, in parallel with the blocking buffer drops. Forty μl is the minimum volume you should use. You can use up to 100 μl per coverslip, but this can be costly if you are using expensive antibodies and staining lots of cultures. Use blocking buffer without antibodies for the –ve controls.
2. To apply primary antibody to the first coverslip, carefully lift it and without washing it in PBS, place it face down on the NF/GFAP antibodies drop. Repeat with the 2^nd^ coverslip, placing it on the NF/IBA1 antibodies drop and so on. Incubate at room temp for 1 hour or overnight at 4^o^C (the latter is preferred, as it increases specificity).
3. Prepare the secondary antibodies in blocking buffer (keep secondary antibodies out of direct light as much as possible):
   1. dilute goat anti-**rabbit** IgG (H+L) secondary antibody, Alexa Flour® 488 conjugate 1 in 1000 **AND** goat anti-**mouse** IgG (H+L) secondary antibody, Alexa Flour® 568 conjugate 1 in 1000 i.e. 1 μl of each in 1000 μl blocking buffer
   2. dilute goat anti-**rat** IgG (H+L) secondary antibody, Alexa Flour® 488 conjugate 1 in 1000 **AND** goat anti-**mouse** IgG (H+L) secondary antibody, Alexa Flour® 568 conjugate 1 in 1000
   3. dilute goat anti-**rat** IgG (H+L) secondary antibody, Alexa Flour® 488 conjugate 1 in 1000 **AND** goat anti-**mouse** IgG (H+L) secondary antibody, Alexa Flour® 568 conjugate 1 in 1000
4. Place 2 x 50 μl drop of each secondary antibody mixture on the staining tray (remember, the negative controls must be incubated in secondary antibody).
5. To **remove excess, unbound primary antibody*** from the first coverslip, carefully lift it (REMEMBER WHICH SIDE IS FACE UP AND TAKE CARE TO AVOID DROPPING THE COVERSLIP) and wash it in PBS by immersing it in each of the three PBS baths, **draining onto tissue between baths**, then place it face down on the first of the secondary antibody drops. Repeat with the 2^nd^ and the 3^rd^ coverslips. Incubate for 60 minutes in the dark at RT. ****Your staining will appear fuzzy if you do not wash your coverslips well. It is extremely important to get rid of excess, unbound antibody. You will not wash off the bound antibody!***
6. Use a **pencil** to label each for your three glass slides with the experiment number, the age of the cultures and the stains used; one with NF(red) and GFAP (green); one with NF (red) andIBA1 (green); one with NF (red) and MBP (green). Place two 5 μl drops of mounting medium with DAPI on each slide. If you are using a mounting medium that sets e.g. Mowiol, do not wait long before mounting the coverslips.
7. To **remove excess, unbound secondary antibody*** lift the 1^st^ coverslip (REMEMBER WHICH SIDE IS FACE UP AND TAKE CARE TO AVOID DROPPING THE COVERSLIP) and wash it 3 x in PBS and then in water (to remove the salt, which would otherwise precipitate on the coverslip) as before. ***Your staining will appear fuzzy if you do not wash your coverslips well. It is extremely important to get rid of excess, unbound antibody. You will not wash off the bound antibody!***
8. Remove the excess water by tapping the edge of the coverslip on blue tissue roll then place it face down on the mounting medium on the slide labelled NF (red) and GFAP (green).
9. Repeat steps 15 and 16 with coverslips 2-6, placing them on the appropriately labelled slides
10. If using a non-setting mounting mediuam, seal the coverslips onto the slides with nail enamel by carefully painting the edges of the slip and **allow the nail enamel to dry** before viewing under the microscope. If using Mowiol, allow to set overnight before viewing slides. This is to prevent contamination of the microscope objectives.
