# Supplementary material for: An in vitro model for studying CNS white matter: functional properties and experimental approaches
Source: F1000Res. 2019 Jan 29;8:117. [Version 1] doi: 10.12688/f1000research.16802.1 (PMC6489523; doi:10.12688/f1000research.16802.1)
Supplement: Supplementary file 6 [file f1000research-8-18368-s0005.tgz › 5cd5f1dd-862a-409f-84f3-205497a2e691_Suplementary_material_5_Protocol_for_using_CellProfiler_software_on_96-well_plate_images.docx]

**Measuring plate In Cell Analyzer 2000**

Measure 4 channels:

DAPI (Hoechst) 350_50 x 455_50 m

FITC (green) 490_20 x 525_36 m

TexasRed (red) 579_34 x 624_40 m

Brightfield

Use:

filter set Quad2

10X magnification

6 fixed fields/well measured

Focus set 7.0 (check for focus in FITC [myelin] channel)

**Image processing using CellProfiler:**

Create a duplicate folder of data and rename the original folder RAW

Use the other folder to rename the images using Bulk Rename Utility (download from <http://www.bulkrenameutility.co.uk>)

Repl(3) window

Replace ( with space

Select files in upper right window, this will show the new name in green and start renaming

**A - 1(fld 1 wv DAPI - DAPI).tif** becomes **A - 1 fld 1 wv DAPI - DAPI).tif**

**How to use CellProfiler:**

Open program

File> import pipeline> from file> select pipeline

Drag input folder into file list box on the right

Under output, select view output settings and select output folder

(eg. for pipeline 1 and 2 use same file as input folder, for pipeline 3 create new output folder)

Run pipeline by hitting Analyze Images (bottom)

*If not responding, drag input folder again in file list box, new files might have been added to the folder with previous pipelines which program has not registered.*

1. Drag the folder with the renamed files into the file list box.

Under output, select input and output folder (same as input folder)

Run quality control pipeline (**1_QCpipeline.cppipe**) this will automatically:

Flag images that do not meet any of the following criteria:

Nuclei count > 1500

Axon area > 40%

PLLS > -2.0

(PLLS is the power log-log slope and indicator of amount of blur in the image. The bigger the slope, the more blur)

Images that pass get 0, images that fail get 1

Images.csv file written in input folder will show results of quality control as well as flagged images

DefaultDB.db and DefaultDB.properties files also written in input folder to use in CellProfiler Analyst

*Also check quality using plateviewer CellProfiler Analyst (use defaultDB.properties file that is written)*

2. In the input file, create a new folder named Sorted

Under output, select input and output folder (output is sorted folder)

Run sort pipeline (**2_sortIMAGESpipeline.cppipe**)

Files with extension _0.tif passed QC, files with extension _1.tif failed QC

3. Drag the folder with the sorted files into the file list box.

Under output, select input and output folder (both sorted folder)

Perform illumination correction calculation (**3_calcILLUMpipeline.cppipe**)

This will create the following files:

AXONILLUM, DAPIILLUM, MYELINILLUM

4. Create a new folder named results

Drag the folder with the sorted files into the file list box.

Under output, select input (sorted folder) and output folder (results folder)

Perform illumination correction and analyse pictures (**4_PROCCESINGpipeline.cppipe**)

This will give you a png file of the resulting images and the excel file **Images** containing:

- Nuclei count
- axon area
- myelin area
- total area
- filename and well info

**How to use CellProfiler Analyst:**

Open program

Select properties file it asks for (in your input folder after running QC pipeline)

**Select plateviewer**

Data source: Per_Image

Measurement:

Choose from the following

Image_AreaOccupied_AreaOccupied_ThreshAxon (impression axon density)

Image_Count_Nuclei (impression cell density)

**Select histogram**

x-as: Per_Image > select parameter you are interested in (e.g. Image_Count_Nuclei) and hit update chart. This will give you impression of spread in data

**Select Scatterplot**

Use this setting to look at overall correlation between different measurements (e.g. nuclei count vs Axon area)
