# Supplementary material for: An in vitro model for studying CNS white matter: functional properties and experimental approaches
Source: F1000Res. 2019 Jan 29;8:117. [Version 1] doi: 10.12688/f1000research.16802.1 (PMC6489523; doi:10.12688/f1000research.16802.1)
Supplement: Supplementary file 2 [file f1000research-8-18368-s0001.tgz › b997fd53-467d-4076-8796-361963d2761e_Suplementary_material_2_Mouse_spinal_cord_cultures_detailed_protocol.doc]

**Mouse spinal cord cultures. Protocol adapted by JME from:**

- Thomson CE, Hunter AM, Griffiths IR, Edgar JM, McCulloch MC (2006) Murine spinal cord explants: a model for evaluating axonal growth and myelination in vitro. J Neurosci Res 84:1703-1715.
- Thomson CE, McCulloch M, Sorenson A, Barnett SC, Seed BV, Griffiths IR, McLaughlin M (2008) Myelinated, synapsing cultures of murine spinal cord--validation as an in vitro model of the central nervous system. Eur J Neurosci 28:1518-1535.

**Solutions:**

Dulbecco’s modified Eagle’s medium (DMEM: Gibco 31885-023) and horse serum ([endotoxin <0.05 IEU for experiments using proteome arrays]) were obtained from Life Technologies, Paisley, UK. Penicillin–streptomycin (P0781), apo-transferrin (T-2252), insulin (I0516 or I2643), putrescine (P7505), sodium selenite (S9133), progesterone (P6149), biotin (B4051), hydrocortisone (H0396), DNase (DN25), poly-L lysine (P1274), and Hank’s balanced salt solution (HBSS; H9394 and H9269) were obtained from Sigma-Aldrich, Dorset, UK.

Use only cell culture grade ingredients – to minimise contamination by endotoxin.

**1. DNAse**

Prepare a 0.5 mg/ml DNase **(Sigma DN25)** solution in DMEM.

DNAse is extremely labile. **Do not freeze-thaw more than once and never vortex. Thaw on ice.** Can be stored **short-term** at 4^o^C after thawing.

*Degrades DNA released from injured cells, which causes viable cells to ‘clump’.*

**2. D-biotin (vitamin H)**

To prepare 10 μg/ml: Resolve biotin **(Sigma B4501)** in 0.01N NaOH to 10 μg/ml and store at 4^o^C.

*Required for carboxylation reactions – important in metabolic pathways.*

**3. Hydrocortisone**

To prepare 10 μM hydrocortisone **(Sigma H0396)**, dissolve in water to 10 mM and store. **Dilute 1 in 1000 in water before adding to differentiation medium**.

Molecular weight is 362.46. Add 1 g to 275 ml water to give 10 mM.

*Required for growth and proliferation of cells. Thomson et al., 2006 found it enhances myelination*

**4. Poly-L-lysine**

Use PLL with molecular weight 70,000 to 150,000. Use 1 in 50 dilution of 5 mg/ml (Sigma Cat no **P1274; which should be frozen in aliquots**) to a final concentration of 0.1 mg/ml (i.e. 0.01%) in boric acid buffer. **Do not freeze the working solution.**

*It is essential that the coverslips/imaging dishes/microelectrode arrays (MEAs) are well-coated. For coverslips or MEAs, place them into a 10 cm Petri-dish containing 20 ml PLL in boric acid buffer. Do not allow them to overlap during incubation. Incubate for at least one hour at room temperature then rinse 2 times with sterile water.*

**5. Boric acid buffer**

1.24g boric acid, 1.9g sodium tetraborate in 350 ml H_2_O, heat to dissolve whilst stirring, pH to 8.5 then top up to 400 ml. Heat or filter sterilse.

*Always prepare PLL in boric acid buffer. It greatly enhances attachment compared to PBS or water but must be washed off carefully! It is highly alkaline.*

**6. 30% glucose**

15 g glucose (**Sigma G7021**) in 50 ml cell culture grade water, filter sterilised and stored at 4^o^C or, in aliquots at -20^o^C.

**7. N1 mix (see below)**

**8. Trypsin** (10x solution - Sigma T4549). Aliquot and freeze in 2.2 ml aliquots.

**9. Coverslips.** VWR Borosilicate glass coverslips, 13 mm diameter, Thickness No. 1, Cat. No. 631-0149, autoclaved. Coat coverslips with PLL in boric acid buffer (BAB) for at least one hour, in 10 cm diameter sterile Petri-dishes. Ensure each coverslip is immersed fully in PLL – do not allow coverslips to lie on top of each other. Rinse thoroughly in sterile filtered water, twice, then apply a further volume of water to the Petri-dish and ‘fish’ out the coverslips using sterile forceps and place 3 into each of several 35 mm Petri-dishes. Place 8 35 mm Petri-dishes inside a 25 cm diameter Petri-dish, and allow coverslips to dry in the fume hood. **Coverslips must be dry before cells are plated onto them.** Place lids on small and large Petri-dishes and store in a sterile bag in the cold room or use immediately. Do not expose coverslips to UV light after coating with PLL.

**10. Live imaging dishes**

Coat dishes with PLL in boric acid buffer (BAB) for at least one hour (~1 ml covering the wells and remaining surface of the Petri-dish) then rinse the dishes in sterile filtered water. *PLL must be rinsed of thoroughly. BAB is pH 8.5!!!* Do not expose dishes to UV light after coating with PLL.

**11. Multi-well plates**

Coat plates with PLL in BAB for at least one hour and rinse of thoroughly with sterile filtered water, using a multi-channel pipette. Plates are 96 [Greiner 655891] or 384-well [Greiner 781856] Sensoplate Plus, black, 175 µM glass bottom, Greiner Bio-One. Do not expose plates to UV light after coating with PLL.

| N1 Mixture |  |  |  |
| --- | --- | --- | --- |
|  | **Weight** | **Solvent Vol** | **Further dilution** |
| Apo-transferrin  Sigma T-2252 | 25 mg | 5 ml PBS  = 5 mg/ml | None |
| Putrescine  Sigma P-7505 | 80.55 mg | 5 ml PBS  = 16.11 mg/ml | None |
| Progesterone  Sigma P-0130  **or** Sigma P-6149 | 3.145 mg | 5 ml 90% ethanol  = 2 x 10^-3^ M | 50 μl to 10 ml PBS  = 10 x 10^-6^ M |
| Selenium (sodium selenite)  Sigma S-9133 | 5.19 mg | 100 ml PBS  =0.0519 mg/ml  or 51.9 μg/ml | 500 μl to 5 ml PBS  =5.199 μg/ml |

**Final N1Mixture**

| Apo-transferrin (*carries iron*) | 5 ml (= 1 mg/ml). Final in medium is 0.005 mg/ml |
| --- | --- |
| Putrescine | 5 ml (= 3.22 mg/ml) which is 20 mM. Final in medium is 0.0161 mg/ml |
| Progesterone | 10 ml (=4 x 10^-6^ M). Final in medium is 2 x 10^-8^ M |
| Selenium (*needed for anti-oxidant enzyme function*) | 5 ml (1.0398 μg/ml) which is 6 μM. Final is 0.005199 μg/ml |

Combine ingredients and filter through 0.22 μm filter. Store at –20^o^C in 0.26 ml volumes.

# PLATING MEDIUM (PM)

Prepare and store in a 50 ml Greiner tube. Cap tightly to make as airtight as possible.

- 50% DMEM (DMEM 31885-023) with pyruvate and phenol red and 5.5 mM D-glucose with added Penicillin/Streptomycin.
- 25% Horse serum (the cells don’t survive if plated without serum).
- 25% HBSS with Ca^2+^ and Mg^2+^

# DIFFERENTIATION MEDIUM

Prepare and store in a 50 ml Greiner tube. Cap tightly to make as airtight as possible.

- 47.9 ml DMEM (Gibco 31885-023) with added Penicillin/Streptomycin
- 0.583 ml 30% glucose (**Sigma G7021**) prepared in advance, filter sterilised and stored at 4^o^C). This makes the final concentration of glucose to 25 mM.
- 0.25 ml 10μM Hydrocortisone (**Sigma H0396**)
- 0.25 ml N1 mix
- 1 ml insulin FOR FIRST 12 DAYS ONLY (**Sigma I2643)** at 0.5 mg/ml in 0.01N HCl (stored at 4 ^o^C) or 200 μl of the same at 2.5 mg/ml (stored at -20^o^C). Alternatively, use 50 μl of **Sigma I0516** at 10 mg/ml OR 0.5 ml of 1 mg/ml solution (**Sigma I6634).**
- 50 μl biotin (Sigma B4501) at 10μg/ml.

**Advance Preparation:**

Use E13 mouse embryos (day of plug = E0). *The age of the embryo is critical*. As a guide, 5 cords will provide enough cells for roughly 30 coverslips at 150,000 cells/coverslip. The relationship between myelination and cell number is not linear – ***myelination depends on a critical cell density and then increases with increasing density*.**

Monitor pregnancy by weighing female from E9 onward. For example, a female carrying 5 foetuses gains 7-8 g by embryonic day 13.

Prepare coverslips, multi-well plates, imaging dishes or MEAs by coating with PLL, as described above. Allow to dry and store at 4^o^C if not required immediately. **Do not expose to UV light once coated with PLL.**

**Evening before:**

1. Book dissecting microscope
2. Sterilise instruments
a) Dissecting scissors and forceps for opening abdominal skin
b) Fine dissecting scissors and fine forceps for opening peritoneum and removing embryos
c) 2 pairs very fine forceps for isolating embryonic cords and removing meninges; coarser forceps for stabilising embryo while dissecting cord; spring scissors for opening uterine sac; sterile scalpel blade for removing head

**Day of experiment:**

**Prepare bench for dissection**

1. Dissecting microscope (booked in advance)
2. Dissecting instruments (as above) and large scissors/forceps for cervical dislocation of dam
3. Sterile 10 cm Petri-dish on ice, lid on
4. 10x trypsin, on ice to thaw
5. ~ 4 ml HBSS **minus** Ca^2+^ and Mg^2+^, on ice
6. 2 x sterile 35 mm Petri-dishes for dissecting embryos and removing meninges
7. 7 ml bijou (e.g. Star Lab E1412-0710) with your initials on the lid and on the side
8. Labelled 0.5 ml tubes, if the embryos/mother need to be genotyped
9. 1 x 100 μl and 1 x 1000 μl pipettes and filter-top tips
10. Sterile scalpel blade and blade handle

**Prepare cell culture suite**

1. Open cell culture hood, turn fan on and spray hood with 70% ethanol
2. ‘Plating media’ and ‘differentiation media plus insulin’ in 50 ml Greiner tubes, at room temperature, upright in a rack
3. DNAse on ice or in fridge to thaw
4. 2 x 13 ml conical tubes, labelled with your initials and Tube1 and Tube 2, plus a balance tube containing water and a rack to hold them upright
5. Haemocytometer and 0.4% trypan blue
6. 35 mm Petri-dishes with coverslips and/or imaging dishes and/or microelectrode arrays (MEAs) coated with PLL in boric acid buffer, inside larger 10 or 25 cm Petri-dishes, labelled with your name and the date and the experiment number.
7. Pipettes, Pipettman, pipette tips, serological pipettes 5 ml syringe and 1 each of 21 and 23 gauge needles

**Kill pregnant dam in specified Schedule 1 room**

Kill pregnant female by cervical dislocation and subsequent decapitation (after having been trained in the method). Transfer mouse to appropriate area of the Schedule 1 room and work quickly to minimise the length of time the mouse is stressed. Alternatively, kill the mouse in increasing concentrations of CO_2_, then decapitate.

Spray abdomen with 70% ethanol and make a nick in the abdomen at the lower midline, using dissecting scissors and forceps. **Tear** open the abdominal skin (tearing reduces contamination from hair *cf* cutting) then cut open the peritoneum using a 2^nd^ pair of forceps and dissecting scissors

Remove uterine horns by lifting up and away from the body (do not allow them to touch the skin, which is not sterile) into an empty sterile 10 cm Petri-dish on ice and transfer to bench

Dispose of carcass appropriately. Check first if other users can use tissues.

**Dissect embryos at the bench**

Pipette 1-2 ml chilled sterile HBSS minus calcium and magnesium into a 35 mm Petri dish on ice.

Working in the 10 cm Petri dish and using the dissecting microscope on low magnification, remove embryos from uterine sac using forceps and spring scissors and cut the head off each one using a scalpel blade at a point **immediately** behind the eye and about 3 mm cranial of the cervical flexure (CF). This means that the medulla is attached to the spinal cord. The medulla is stronger than the cord and provides a useful site for stabilising with forceps. The liquid released when the uterine sacs are opened is sufficient to keep the embryos moist during the dissection.

CF

Dorsal aspect of dissected cord is open along the midline

Side on view of embryo

Position the embryo with dorsal aspect up (i.e. face down) and use one point of sharp forceps to ‘cut’ the skin and the meninges overlying the dorsal vein (runs right up the spinal cord), from the base of the spine to the head, while stabilising the body at the hips with blunt forceps. To do this, anchor the body at the level of the hips with the forceps in your left hand and use the point of the forceps in your right hand to ‘cut’ up the midline of the cord from the hips to the neck. Then use the forceps in you right hands like scissors to transect the cord just above the level of the hips. Close these forceps and then use them to lift and roll the cord up (towards the head) and out of the body, while stabilising the body at the level of the hips with the forceps in your left hand. Lift the cord at the medulla with forceps, place it into a clean 35 mm Petri dish with 1-2 ml chilled HBSS minus calcium and magnesium (trypsin only works in the absence of Ca^2+^ and Mg^2+^ ions). Repeat for all embryos.

With the cord dorsal side up, grab the meninges at the cervical region of the cord (where they are attached loosely) while holding onto the medulla with the forceps in your left hand and pull the meninges off as if you were pulling off a sock. Removal of the meninges will eliminate most of the contaminating PNS material; still, it is likely that the cultures will contain some Schwann cells and DRGs. Repeat for all cords.

Using forceps, transfer cords to 1 ml HBSS (without Ca^2+^ and Mg^2+^) in a 5 ml bijou, on ice. If you have more than 6 cords, use 2 ml HBSS. When you have finished collecting the cords, add 100 μl (200 μl if > 6 cords) 10 x trypsin. Pipette up and down **gently** using a 1 ml pipette tip to break cords into small pieces – this increases the surface area for access by trypsin. Take bijou to cell culture and incubate at 37^o^C for 15-20 minutes.

**Cell Culture suite**

Spray 7 ml bijou with ethanol (ensure lid is tight first) and transfer the clean, labelled bijou to the cell culture incubator and incubate at 37^o^C for 15-20 minutes.

While tissue is incubating, clean up the dissection area

If you collected tails/ear clips for genotyping, put them on the thermocycler in lysis buffer (preferred) or freeze them

Remove bijou from incubator and place in a rack in the cell culture hood. Stop the trypsin reaction by adding 1 ml plating media (PM) plus 80 μl of DNAse at 0.5 mg/ml to the trypsin/HBSS mixture. (Add 2 ml PM and 160 μl of DNAse at 0.5 mg/ml if 2 ml of trypsin/HBSS was used).

Dissociate the trypsinised material by triturating gently through a 21-gauge needle 4 times, then through a 23-gauge needle 2 times (attached to a 5 ml syringe). Do not over triturate the cells – you will injure them! On the 2^nd^ use of the 23-gauge needle, dispense the cell suspension into a 15 ml centrifuge tube in a rack.

Add 5 ml PM to the cell suspension and spin the cells at ~**800 rpm** on a benchtop centrifuge for 5 minutes. **Never spin higher than 1000 rpm** – you could ‘smash’ the cells! Transfer the supernatant using a serological pipette to the second 15 ml tube and spin again. Meantime, add 1 ml PM to the cell pellet in tube 1 and disperse the cells gently through the medium using a 1 ml pipette tip. Remove the supernatant from tube 2 with a serological pipette and combine the two cell pellets in 2-4 ml PM total, for 6-10 cords.

Adhere the coverslip to a haemocytometer for cell counts. Dilute 10 μl cell suspension (after mixing it thoroughly) in 10 μl trypan blue and pipette cell suspension onto both sides of the haemocytometer.

Using the 10x objective on an inverted microscope, count cells on North and West borders of the counting square (which fills almost the entire field of view) on one side of the haemocytometer. Do not count cells on S and E borders. If you have fewer than 100 cells per **counting** square, your count is unlikely to be accurate. Count cells on the opposite side of the haemocytometer. **Do not count blue cells – they are dead!**

Take the average of the two counts. If they are very different, it suggests you have not mixed the cells well so sample again. Multiply the average by 2 to take account of dilution by trypan blue. Multiply by number of ml of cells suspension. Multiply by 10^4^ to give you the total number of cells.

e.g. 140 + 160 = 300. 300/2 = 150 cells on average. 150 x 2 (dilution by trypan blue) = 300. 300 x 4 (number of mls of cells) = 1200. 1200 x 10^4^ = 12,000,000

Using the value obtained, divide by 150,000 i.e. the number of cells to be plated on each coverslip*. For example, 12,000,000/150,000 = 80. You have enough cells for 80 coverslips at 150,000 cells/coverslip. Reduce this by 10-20 % = 72 coverslips. Make volume to 7.2 ml and plate 100 μl of cells suspension on each coverslip (3 coverslips per 35 mm Petri dish), after mixing thoroughly. Ten embryos provide enough cells for ~ 100 coverslips. If your count suggests >100 coverslips, it is probably wrong.

*For imaging dishes, plate 80 μl of cells at the same concentration as above (i.e. 1,500,000 cells per ml) on each well.

*For multi-well plates, make the concentration to 1,000,000 cells per ml and plate 100 or 50 μl per well, into 96 or 384 well plates, using a reservoir and a multi-channel pipette.

***Troubleshooting.*** *In the case of the coverslips, the cell suspension will form a droplet on the coverslip. If it does not and instead spreads out and over the edges of the coverslip, the PLL coating has failed for some reason.*

*Did you allow the coverslips to dry before plating the cells? This is critical.*

*Did you freeze the PLL after it was made to its working concentration? This should never be done.*

After at least two hours, examine the cells on an inverted microscope. Most should have adhered to the coverslips and the cell density in the centre of the coverslip should be high – cells almost touching each other. Add a further 200 μl warmed plating medium (to make a total of 500 μl) to the Petri dish and disperse over the surface of the dish with a pipette tip. Try to wet the edges of the coverslips or they will float later, resulting in sloughing-off of cells. At end of the day feed the cells by adding 500 μl warmed differentiation medium to each Petri-dish. It is possible to do this in a single step, by combining appropriate volumes of the two media and adding 700 μl media to each 35 mm Petri-dish.

Feed cells Mon, Wed and Fri by removing 0.48 ml medium and adding 0.52 ml fresh medium (the difference is the ‘Angel’s share’). Maintain for at least 23 days. Myelination begins around day 17. Cultures can be maintained until 26-28 days and sometimes longer. Examine cells on the inverted microscope before feeding. Check for morphology, viability and contamination.

To add an experimental agent to the 35 mm *Petri-dishes, first dilute it to 2x the required concentration in feeding medium and warm to 37^o^C, then remove 0.48 ml medium from each Petri-dish and add 0.52 ml fresh medium plus experimental agent.

*For multi-well plates, we treat a minimum of 6 wells with a single agent at a single concentration; although initial screens of multiple factors/concentrations can be run with fewer. Make 1000 μl of 2x the required concentration of each factor of interest in feeding medium (e.g. 2 x 1, 2 x 5, 2 x 10, 2 x 50 or 2 x 100 ng ml^-1^ Activn A or 2 x 100 ng ml^-1^ FGF9) and disperse in a pre-generated random fashion into a fresh multi-well plate, the ‘feeder’ plate. It is important to distribute each factor, including controls, randomly across the plate to prevent effects due to uneven heating/cooling/evaporation from influencing the results. The ‘feeder’ plate can be stored at -20^o^C for several days, depending on the stability of the agent. Thaw at 4^o^C and then warm to room temperature for ~30 minutes. Remove ~90 μl per well from the cells and using the multi-channel pipette, dispense ~110 μl per well of factors of interest from the ‘feeder’ plate.

If the factor of interest has to be dissolved initially in a solvent such as DMSO, be sure to include the solvent alone as one of your controls.

To fix the cells, warm 8% paraformaldehyde (PFA) to 37^o^C and add 1 ml each *Petri-dish, to give a final concentration of 4% PFA. Fix for 10 minutes then wash off thoroughly in room temperature PBS. Store cells in PBS (1% PFA in PBS for long-terms storage) or proceed to immunostaining.

*For multi-well plates, first remove 100 μl medium per well, then add 100 μl of warm 8% PFA, using a multi-channel pipette. For antibody staining of multi-well plates, we recommend using a ‘feeder’ plate, as described above, from which to administer antibodies.

***Troubleshooting***

*If the cultures gradually become unreliable, make fresh ingredients - in particular, biotin, hydrocortisone and N1.*

*If you do not see neuritic outgrowth in the first days of the culture, it is unlikely the cultures will work – you have lost the neuronal precursors through over-trituration, inadequate adhesion, failure of DNAse after trituration, or because the cell culture media is no longer viable.*

*If you do not observe myelination, it is likely the neuritic density is too low, so plate cells at a slightly higher density. However, too high a density leads to inadequate diffusion of oxygen and nutrients.*

Keep a record like this one for every cell culture:

| Example Exp # 1  Mouse strain  C57BL/6 | **Mon** | **Tue** | **Wed** | **Thu** | **Fri** | **Sat** | **Sun** |
| --- | --- | --- | --- | --- | --- | --- | --- |
| Week beginning | Feeding | | | | | | |
| 12 Nov 2018 |  | DIV0 |  |  | DifMed+ |  |  |
| 19 Nov 2018 | DifMed+ |  | DifMed+ |  | DifMed+ |  |  |
| 26 Nov 2018 | DifMed- |  | DifMed- |  | DifMed- |  |  |
| 12 Nov 2018 | DifMed- |  | DifMed- |  | DifMed- |  |  |
| 3 Dec 2018 | Fix |  |  |  |  |  |  |

Notes: Record any deviation from the protocol, number of embryos, numbers of cells, when changed to differentiation medium minus insulin (DifMed-), when media and ingredients were prepared.
