# Supplementary material for: An in vitro model for studying CNS white matter: functional properties and experimental approaches
Source: F1000Res. 2019 Jan 29;8:117. [Version 1] doi: 10.12688/f1000research.16802.1 (PMC6489523; doi:10.12688/f1000research.16802.1)
Supplement: Supplementary file 3 [file f1000research-8-18368-s0002.tgz › 59a8e7bd-9c17-4882-95e8-97ad30cb2c0f_Suplementary_material_1_Dishes_for_live_imaging.doc]

**Dishes for live imaging.**

You would need:

- 35 mm Petri dishes (Falcon Ref. 353001 are resistant to cracking).
- 25 mm coverslips (Menzel-Glaser 0,17 +/- 0,01 mm; Starke 1,5; Lot #004710182 from Thermo Scientific) treated for at least 1 hour in nitric acid to etch the coverslips (helping cells adhere) then rinsed very thoroughly in water
- Roper Whitney XX hand punch (Roper Whitney #135010001 with bench mounting base #139010001) to make holes in the bottom of plastic dishes.
- Hand deburring tool (Small Parts Inc #DBR-HT)
- Sylgard 184 glue (World Precision Instruments, #SYLG184) or RTV615 (Momentive [now Hexion]) to glue glass coverslips to dishes.

1. Make 3 x 11 mm diameter holes in the base of a Petri-dish, positioned in such a way that the 25 mm diameter glass coverslip will simultaneously cover all three holes. The holes must be absolutely deburred, otherwise the coverslip will not rest. Take a countersink and deburr the holes by hand.

2. The dish must be degreased (ethanol) before gluing the coverslip

3. After mixing the glue parts together (in a glass vessel), remove the bubbles by vacuum.

4. Apply glue to the base of the Petri dishes using a 21 Gauge needle, bent at a 90^o^ angle

4. Using forceps, place a coverslip onto the glued surface. Do not exert any pressure on the cover slip; its tare weight is sufficient for optimal silicone distribution to generate a continuous adhesive layer.

6. After gluing all dishes, put them in a heat cupboard for 4 hours at 65^o^C or according to manufacturer’s instructions. Allow to cool at room temperature and leave for glue to cure fully for at least 24 hours.

7. Wash dishes briefly (< 1 minute) in 0.02% SDS in water to remove any grease, and **rinse thoroughly at least 3 times in a large volume of running tap water, in a > 1 litre beaker**. Rinse with distilled water, remove excess water onto tissue paper, spray dishes with 70% ethanol and UV sterilise and dry for ~ 1 hour in the cell culture hood.

*If SDS is not rinsed off properly, your cells will die. It is a detergent!!!*

Thanks to the Fine Mechanics at the Max Planck Institute of Experimental Medicine, Goettingen, Germany, for optimising this protocol, which was obtained from users of CONFOCALMICROSCOPY@LISTS.UMN.EDU

Originally from: D. Kline in Microinjections: Methods and applications, ed. DJ Carroll, 2009 Humana Press, pp 135-156.
