# Supplementary material for: An in vitro model for studying CNS white matter: functional properties and experimental approaches
Source: F1000Res. 2019 Jan 29;8:117. [Version 1] doi: 10.12688/f1000research.16802.1 (PMC6489523; doi:10.12688/f1000research.16802.1)
Supplement: Peer review history

# Peer review: Reviewer response for version 1

**DOI:** 10.5256/f1000research.18368.r43885

**Published:** 2019-02-19

**Authors:** Jacqueline Trotter

**Affiliations:** 1 Department of Biology, Molecular Cell Biology, Institute of Developmental Biology and Neurobiology, Johannes Gutenberg University of Mainz, Mainz, Germany

This article expands in substantial detail on a previously published method of myelinating cultures generated from murine spinal cord. The authors clearly demonstrate that establishing the cultures on MEA chips facilitates the monitoring of electrophysiological properties of the system. They show clearly the manipulation of gene expression in the culture using siRNA and also the ability to study developmental and pathological processes, such as innate immune responses, using imaging. They also demonstrate the use of multi-well plates facilitating drug screening.

 The article thus describes an excellent straightforward method to complement, and in some instances replace, experiments in live animals. The method will be very useful for screening reagents with the potential to influence myelination or remyelination. It therefore provides a timely and important contribution to the experimental toolbox of myelin researchers.

**Are a suitable application and appropriate end-users identified?**

 Yes

**If applicable, is the statistical analysis and its interpretation appropriate?**

 Yes

**Are the 3Rs implications of the work described accurately?**

 Yes

Is the rationale for developing the new method (or application) clearly explained?

Yes

Is the description of the method technically sound?

Yes

Are the conclusions about the method and its performance adequately supported by the findings presented in the article?

Yes

If any results are presented, are all the source data underlying the results available to ensure full reproducibility?

Yes

Are sufficient details provided to allow replication of the method development and its use by others?

Yes

Reviewer Expertise:

Myelination, primary cell culture, siRNA

I confirm that I have read this submission and believe that I have an appropriate level of expertise to confirm that it is of an acceptable scientific standard.

# Response

**Published:** 2019-02-19

**Authors:** Julia Edgar

**Affiliations:** University of Glasgow, UK

We are very grateful to reviewer for taking the time to carefully read and consider our manuscript, which became rather long in the end.

# Peer review: Reviewer response for version 1

**DOI:** 10.5256/f1000research.18368.r43736

**Published:** 2019-02-14

**Authors:** Tracey A. Newman

**Affiliations:** 1 Southampton Neuroscience Group, Institute for Life Sciences, Centre for Biological Sciences, University of Southampton, Southampton, UK

The disease burden associated with nervous system white matter (myelin) changes is significant. Newer imaging modalities/image analyses are enabling the detection of white matter changes in the clinic. The upshot of this is that there is a need for robust systems for the study of the mechanisms underlying demyelination/dysmyelination and systems in which to study remyelination. A significant amount of the research to address these issues relies on the use of in-vivo rodent experiments. Many of which fall into the moderate category of in-vivo procedure at a minimum. This work describes a relatively straight-forward model system which could replace, or be used to inform, some of the in-vivo work.

 The rationale is clear and the methods are thoroughly described. The description and interpretation of the data is robust, and the suggested utility of the method is proportionate. The manuscript would be improved further by considering the following:

  

1. A short section describing similar/or equivalent in-vitro systems ideally describing the advantage of the system described.
2. Clarification regarding the value of having a system that is not part of a whole system (in the research highlights this is presented as a strength, but why is not made explicit).
3. The cultures have been exploited for a number of features, and this is supported by the data that is presented – however some analysis of the cellular organisation (2D/3D) of the cultures and the variability in this would be very useful as other researchers consider whether the system is appropriate for the experimental questions that want to address using the culture. It would also enable the myelination to be seen in a different orientation, and support the data described. One approach to this would be to section the cultures, possibly using an en-face approach as the cultures are grown on coverslips. This approach has been used in other organoid/mini-brain culture analyses.
4. A key issue with the development of new methods is the dissemination to other groups, the authors report that the model could be readily adopted in other labs equipped for cell culture. Has this happened? Irrespective of whether the model has been used to test a hypothesis elsewhere, it would be useful to know that a second group working from the protocols has reported similar findings, this will increase confidence in the model.
5. Re the methods, describe briefly the sterilisation step for the live imaging dishes.
6. Minor points a careful final proof-read is needed e.g. flurobite p5 needs to be corrected.

**Are a suitable application and appropriate end-users identified?**

 Yes

**If applicable, is the statistical analysis and its interpretation appropriate?**

 Yes

**Are the 3Rs implications of the work described accurately?**

 Yes

Is the rationale for developing the new method (or application) clearly explained?

Yes

Is the description of the method technically sound?

Yes

Are the conclusions about the method and its performance adequately supported by the findings presented in the article?

Partly

If any results are presented, are all the source data underlying the results available to ensure full reproducibility?

Yes

Are sufficient details provided to allow replication of the method development and its use by others?

Yes

Reviewer Expertise:

Referee suggested by the NC3Rs for their scientific expertise and experience in assessing 3Rs impact

I confirm that I have read this submission and believe that I have an appropriate level of expertise to confirm that it is of an acceptable scientific standard.

# Response

**Published:** 2019-02-19

**Authors:** Julia Edgar

**Affiliations:** University of Glasgow, UK

We are very grateful to the reviewer for taking the time to carefully read and consider the manuscript and will respond more fully to her comments shortly.
